# Effect of high-dose *Spirulina* supplementation on hospitalized adults with COVID-19: a randomized controlled trial

**DOI:** 10.3389/fimmu.2024.1332425

**Published:** 2024-04-08

**Authors:** Mohammad Reza Aghasadeghi, Mohammad Ali Zaheri Birgani, Saeedreza Jamalimoghadamsiyahkali, Hadiseh Hosamirudsari, Ali Moradi, Majid Jafari-Sabet, Nooshin Sadigh, Pooneh Rahimi, Rezvan Tavakoli, Mojtaba Hamidi-Fard, Golnaz Bahramali, Zohal Parmoon, Sina Arjmand Hashjin, Ghasem Mirzajani, Reza Kouhkheil, Somayeh Roshangaran, Samineh Khalaf, Mohammad Khademi Nadoushan, Ghazaleh Gholamiyan Yousef Abad, Nima Shahryarpour, Mohammad Izadi, Abolfazl Zendedel, Shayesteh Jahanfar, Omid Dadras, SeyedAhmad SeyedAlinaghi, Daniel Hackett

**Affiliations:** ^1^ Hepatitis and AIDS Department, Pasteur Institute of Iran, Tehran, Iran; ^2^ Viral Vaccine Research Center, Pasteur Institute of Iran, Tehran, Iran; ^3^ Iranian Research Center for HIV/AIDS, Iranian Institute for Reduction of High-Risk Behaviors, Tehran University of Medical Sciences, Tehran, Iran; ^4^ Ziaeian Hospital, Tehran University of Medical Sciences, Tehran, Iran; ^5^ Department of Infectious Disease, Baharloo Hospital, Tehran University of Medical Sciences, Tehran, Iran; ^6^ Department of Pharmacology, School of Medicine, Iran University of Medical Sciences, Tehran, Iran; ^7^ Laboratory Department, Baharloo Hospital, Tehran University of Medical Sciences, Tehran, Iran; ^8^ Emergency Department, Baharloo Hospital, Tehran University of Medical Sciences, Tehran, Iran; ^9^ Intensive Care Unit, Baharloo Hospital, Tehran University of Medical Sciences, Tehran, Iran; ^10^ Department of Internal Medicine, Ziaeian Hospital, Tehran University of Medical Sciences, Tehran, Iran; ^11^ Family Medicine Department, Ziaeian Hospital, Tehran University of Medical Sciences, Tehran, Iran; ^12^ Department of Public Health and Community Medicine, Tufts University School of Medicine, Boston, MA, United States; ^13^ Physical Activity, Lifestyle, Ageing and Wellbeing Faculty Research Group, School of Health Sciences, Faculty of Medicine and Health, The University of Sydney, Sydney, NSW, Australia

**Keywords:** *Spirulina platensis*, COVID-19, intensive care unit (ICU) & non-ICU, mortality, hospital discharge, immune mediators

## Abstract

**Objective:**

*Spirulina* (*arthrospira platensis*) is a cyanobacterium proven to have anti-inflammatory, antiviral, and antioxidant effects. However, the effect of high-dose *Spirulina* supplementation on hospitalized adults with COVID-19 is currently unclear. This study aimed to evaluate the efficacy and safety of high-dose *Spirulina platensis* for SARS-CoV-2 infection.

**Study Design:**

We conducted a randomized, controlled, open-label trial involving 189 patients with COVID-19 who were randomly assigned in a 1:1 ratio to an experimental group that received 15.2g of *Spirulina* supplement plus standard treatment (44 non-intensive care unit (non-ICU) and 47 ICU), or to a control group that received standard treatment alone (46 non-ICU and 52 ICU). The study was conducted over six days. Immune mediators were monitored on days 1, 3, 5, and 7. The primary outcome of this study was mortality or hospital discharge within seven days, while the overall discharge or mortality was considered the secondary outcome.

**Results:**

Within seven days, there were no deaths in the *Spirulina* group, while 15 deaths (15.3%) occurred in the control group. Moreover, within seven days, there was a greater number of patients discharged in the *Spirulina* group (97.7%) in non-ICU compared to the control group (39.1%) (HR, 6.52; 95% CI, 3.50 to 12.17). Overall mortality was higher in the control group (8.7% non-ICU, 28.8% ICU) compared to the *Spirulina* group (non-ICU HR, 0.13; 95% CI, 0.02 to 0.97; ICU, HR, 0.16; 95% CI, 0.05 to 0.48). In non-ICU, patients who received *Spirulina* showed a significant reduction in the levels of IL-6, TNF-α, IL-10, and IP-10 as intervention time increased. Furthermore, in ICU, patients who received *Spirulina* showed a significant decrease in the levels of MIP-1α and IL-6. IFN-γ levels were significantly higher in the intervention group in both ICU and non-ICU subgroups as intervention time increased. No side effects related to *Spirulina* supplements were observed during the trial.

**Conclusion:**

High-dose *Spirulina* supplements coupled with the standard treatment of COVID-19 may improve recovery and remarkably reduce mortality in hospitalized patients with COVID-19.

**Clinical Trial Registration:**

https://irct.ir/trial/54375, Iranian Registry of Clinical Trials number (IRCT20210216050373N1)

## Introduction

Multiple human cases of a novel coronavirus infection called severe acute respiratory syndrome coronavirus 2 (SARS-CoV-2) were first reported in Wuhan, China (December 2019) and have caused the global outbreak of COVID-19 (1, 2). The majority of COVID-19 patients exhibit mild symptoms and do not require hospitalization. However, approximately 30% of adult patients experience acute respiratory distress syndrome (ARDS) and severe pneumonia, leading to potential systemic complications and an increased mortality risk that necessitates critical care (3–5). Despite the positive effects of antiviral and anti-inflammatory treatments, supportive therapy remains the primary management strategy for this disease due to the absence of a definitive treatment for COVID-19 (6–8).

SARS-CoV-2 infection triggers the innate immune system, leading to the release of various types of cytokines, a phenomenon known as a “cytokine storm.” This results in a systemic inflammatory response and can cause multiple organ failure. Prior studies on the innate immune response in COVID-19 patients have noted an increase in total neutrophils, serum interleukin-6 (IL-6), and C-reactive protein (CRP), along with a decrease in total lymphocytes (9–12). Severe COVID-19 patients exhibit uncontrolled production of inflammatory mediators, leading to organ dysfunction. SARS-CoV-2 causes pulmonary dysfunction, worsening arterial hypoxemia and leading to ARDS (9, 10, 13). Macrophage activation triggers an excessive immune response in these patients (9, 10, 14). Increased cytokines and biomarkers like IL-6, tumor necrosis factor-alpha (TNF-α), interferon gamma-induced protein 10 (IP-10), ferritin, and CRP exacerbate ARDS. TNF-α and IL-6 induce acute phase proteins and inflammation. Studies show a positive correlation between IL-6 and CRP levels (9–12, 15). A rapid increase in CRP predicts respiratory deterioration. High IL-6 levels are associated with pulmonary progression, while decreasing IL-6 levels improve it (10, 11, 15). Tocilizumab-treated patients, an IL-6 receptor monoclonal antibody, show a rapid decrease in CRP levels (15). Changes in immune mediators, especially reduced IL-6 levels, seem to reduce inflammatory biomarkers and improve oxygen saturation.

A prospective study on SARS indicated that a decrease in SARS-CoV load precedes disease progression, suggesting that excessive immune responses, rather than uncontrolled viral replication, may be responsible for disease progression and subsequent lung damage (16). Therefore, it is reasonable to suggest that treating COVID-19 patients solely with antiviral medication may not be the most effective approach.

Dietary supplements have been reported to have a positive effect on viral infections (17, 18). *Spirulina* (*arthrospira platensis*), a filamentous, gram-negative cyanobacterium, is a blue-green microalga that is a non-nitrogen-fixing photoautotroph (19). *Spirulina platensis* is rich in protein (over 70%), vitamins, and minerals such as vitamin D, B12, provitamin A (beta carotene), and iron. It also contains phenolic acids, tocopherols, γ-linolenic acid, and is particularly high in phycocyanobilin (PCB). PCB, a blue pigment protein, is part of the light-harvesting phycobiliprotein family and has anti-inflammatory, anti-cancer, and antioxidant properties. *Spirulina platensis*, which does not contain cellulose, is easily digestible (20–22). Previous studies have confirmed the anti-inflammatory properties of *Spirulina platensis*, demonstrating its ability to prevent and reduce inflammation by inhibiting histamine release from mast cells (20, 23, 24). Research shows that *Spirulina* consumption increases B-group vitamins, particularly B6, and decreases interleukin-4 (IL-4) levels in individuals with allergic rhinitis. It also increases immunoglobulin A levels in saliva, thereby enhancing mucosal immunity (20, 21, 25, 26). *Spirulina platensis* boosts the function of natural killer (NK) cells and increases the secretion of interferon-γ (IFN-γ), thereby improving the innate immune system (20, 21, 27). *Spirulina* also contains an effective substance called calcium spirulan (Ca-Sp), which, according to *in vitro* studies, can inhibit the proliferation of various enveloped viruses, including mumps virus, measles virus, influenza A virus (IAV), human immunodeficiency virus type 1 (HIV-1), human cytomegalovirus (CMV), and herpes simplex type 1 (HSV-1) (28–31).

Previous studies suggest that macrophages, not directly infected by SARS-CoV-2 is causal in the cytokine storm seen with COVID -19 patients (9, 10, 14). The mechanism is unclear, but IFN-I signaling, produced by SARS-CoV-2 infected plasmacytoid dendritic cells (pDCs), is linked to this storm (32). The noncanonical NF-κB (nuclear factor kappa-light-chain-enhancer of activated B cells) pathway, a key mediator of inflammatory responses, controls IFN-I production (32). NF-κB activation in various cell types leads to pro-inflammatory cytokines production (9, 33). Studies suggest inhibiting the NF-κB pathway could potentially be effective in treating COVID-19. *Spirulina* has been shown to inhibit the NF-κB pathway and induce Nrf2 (nuclear factor erythroid 2–related factor 2) activation (34–39).

*Spirulina* possesses anti-inflammatory, antiviral, and antioxidant properties. However, its impact on COVID-19 patients remains unverified. To assess the effectiveness and safety of high-dose *Spirulina platensis* in treating SARS-CoV-2 infection, we carried out a randomized, controlled, open-label trial involving adult patients who were hospitalized due to COVID-19.

## Materials and methods

### Patients

Patients were eligible to participate if they were hospitalized with the clinical diagnosis of COVID-19. This diagnosis needed to be confirmed via a positive PCR test, chest CT scan results, and, finally, the definitive diagnosis of the attending physician. Patients also needed to be ≥18 years old and have an oxygen saturation level of ≤94%. All patients gave written informed consent before starting the study. Patients were excluded if they had renal failure, were pregnant or breastfeeding, required intubation on admission, or had cancer, liver dysfunction, multiple sclerosis, lupus, rheumatoid arthritis, or phenylketonuria. Additionally, participants were excluded if they took warfarin, opium, immunosuppressants, or were allergic to microalgae. Between June 2021 and February 2022, 429 patients were screened for eligibility, and 72 were not eligible. The main reasons for ineligibility included oxygen saturation >94%, negative PCR test, and arbitrary use of opium as a treatment for COVID-19. Of 357 eligible patients, 111 did not consent to the study, and 57 participants withdrew their consent within the first 24 hours (non-ICU: *Spirulina* (n=16), control (n=14); ICU: *Spirulina* (n=15), control (n=12).

### Study design

A randomized controlled trial study design was used to examine the research question with patients and medical staff unblinded to the treatment (open-label trial). This study was a multicenter trial involving both Ziaeian Hospital and Baharloo Hospital, which are affiliated with the Tehran University of Medical Sciences in Tehran, Tehran Province, Iran. Patients were recruited from June 27, 2021 (the date of enrollment of the first patient) to February 17, 2022 (the date of enrollment of the last patient). Initially, this study was a single-blind trial; however, there was a negative attitude toward the placebo (the patients did not trust the placebo, and none of the patients participated in the study) among Iranian patients, which led to changing the study method and removing the placebo. Placebo was removed from the study protocol on June 19, 2021. Eligible patients (intensive care unit (ICU) and non-ICU) were randomly divided in a 1:1 ratio to receive 15.2 g (ALGOTAB Algae Powder Capsules) of oral *Spirulina* supplement daily plus standard treatment COVID-19 (*Spirulina* group) or standard treatment COVID-19 alone (control group) for six days. Post-hospitalization screening was conducted for non-ICU patients, while screening for ICU patients was done upon their admission to the ICU to determine their eligibility for study participation. Standard treatment for COVID-19 was consistent with the latest version of these guidelines. Briefly, this included antiviral therapy (remdesivir), corticosteroids (dexamethasone), and anticoagulants (heparin or enoxaparin). In patients who, despite receiving the standard treatment for COVID-19, within 24-72 hours of admission, the course of the disease progressed, and CRP ≥75, treatment was done with tocilizumab. In accordance with the national protocol at the time, methyl prednisolone was utilized for maximum of three days as an alternative when tocilizumab was either unavailable or contraindicated. Additionally, if respiratory support was needed, necessary measures (IMV, NIV, and HFNC) were taken. Randomization was done for each center separately, and the randomization sequences were generated using the Random Allocation Software^©^ (RAS; Informer Technologies, Inc.). Also, considering the two groups of this study and the method of this study (open-label trial), randomization was done using blocks with variable sizes of 4, 6, or 8 patients. The randomization of patients was classified based on the Ward of hospitalization (ICU and non-ICU), which is a measure of the severity (severe and critical severity) of the disease. In order to avoid conscious and unconscious bias of researchers and medical staff, allocation concealment was done using the central randomization method. In this way, after checking the patients’ eligibility to enter the study and obtaining informed and written consent from the patients, each center contacted the designated center (Iranian Research Center for HIV/AIDS) by phone to allocate the participants to the study groups; eventually, the patients were randomly allocated to the study groups (*Spirulina* and control group). The trial was conducted according to the Declaration of Helsinki and the Iran Ministry of Health requirements for clinical trials and was approved by the Research Ethics Committee of Tehran University of Medical Sciences (IR.TUMS.IKHC.REC.1399.481) and the Iranian Registry of Clinical Trials (IRCT) registry team. The study protocol is registered with IRCT under IRCT20210216050373N1, available at https://irct.ir/trial/54375.

### Clinical and laboratory monitoring

Progress or improvement of the disease in terms of clinical symptoms and clinical and laboratory findings for each patient was recorded in the daily assessment form (DAF). Finally, the data from days 1, 3, 5, and 7 were collected using DAF. A blood sample of 10 ml was taken from all patients on days 1, 3, 5, and 7 to check immunological tests (levels of cytokines and chemokines). Plasma separation from whole blood samples was done < 2 hours after sampling and stored in a -80°C freezer. Cytokines and chemokines levels for patients were measured by enzyme-linked immunosorbent assay (ELISA) on plasma samples according to the manufacturer’s recommendations (Abcam, IL-6, ab178013; IP-10, ab173194; interleukin-10 (IL-10), ab100549; TNF-α, ab181421; IFNγ, ab46025; monocyte chemotactic protein 1 (MCP-1), ab179886; macrophage inflammatory protein 1α (MIP-1α), ab214569). The samples collected for the non-ICU subgroup are as follows: *Spirulina* group (first day n=44, third day n=44, fifth day n=41, and seventh day n=23) and control group (first day n=46, third day n=46, fifth day n=46, and seventh day n=38). As for the ICU subgroup, samples collected are as follows: *Spirulina* group (first day n=47, third day n=47, fifth day n=40, and seventh day n=38); control group (first day n=52, third day n=48, fifth day n=45, and seventh day n=41). To avoid the reduction of the study population on the seventh day in the non-ICU, the patients who were discharged from the hospital on the sixth day were asked to return to the hospital for the final sampling. Ten patients in the *Spirulina* group and seven in the control group were discharged on the sixth day. The same dosage of *Spirulina* supplement on the sixth day was given to the patients in the *Spirulina* group who were discharged.

### Outcome measures

Primary endpoints included mortality and hospital discharge within seven days of randomization. The discharge of the participants from the hospital was based on the physician’s decision. Specifically, the absence of shortness of breath or fever, improvement in fatigue and cough, tolerance of oral feeding, and above all, stable oxygen saturation of ≥95%. Secondary endpoints included mortality and hospital discharge overall, from the time of hospitalization to death or hospital discharge. Additionally, transferring patients from ICU to Ward and from Ward to ICU was considered an outcome of recovery or non-recovery. Furthermore, in this study, headache, fatigue, sore throat, cough, temperature, and oxygen saturation were considered subsidiary clinical outcomes.

### Statistical analysis

In order to achieve a power level of at least 90% and a two-sided significance level of α=0.05, it was calculated that 96 patients would need to be assigned to each group. This calculation assumes a 20% dropout rate, resulting in a total of 120 patients per group and 240 patients overall. The sample size was calculated using G*Power software version 3.1.9; no interim analysis was planned. Numerical variables were described based on the distribution as median with interquartile range (IQR) or mean with standard deviation (SD); these values were compared with a t-test or Mann-Whitney U test. The paired t-test or Wilcoxon matched-pairs signed rank test was used to compare immune mediators before and after treatment in the study groups based on the normal distribution test (Shapiro-Wilk test and Kolmogorov-Smirnov test). Categorical data were described in frequency and number and compared with the chi-square or Fisher’s exact tests. For non-numerical variables, primary and secondary outcomes, hazard ratio (HR, Mantel-Haenszel), and 95% confidence interval (CI) were estimated. Due to the lack of events in some variables, such as the death variable in the intervention group, the Mantel-Haenszel method was used to calculate the hazard ratio; in cases where the GraphPad Prism software calculates the confidence interval for a parameter, it can be concluded that there is scientific logic in calculating the estimate of that parameter.

Furthermore, for the numerical variables of the outcomes, the differences were expressed based on the distribution in the form of median difference (Hodges–Lehmann estimate), mean difference, and 95% CI. To better understand whether clinical treatment was effective or not, all non-numeric variables were expressed as the number needed to treat (NNT) with absolute risk reduction (ARR) or the number needed to harm (NNH) with absolute risk increase (ARI) and 95% CI (29). Kaplan-Meier analysis was used to estimate mortality, hospital discharge, and transfer of patients between hospital Wards during the 7-day follow-up and total length of hospitalization to reflect differences between the *Spirulina* and control groups. The extracted curves were compared with the Log-rank (Mantel-Cox) test. Efficacy analyses were conducted on an intention-to-treat (ITT) basis, hence including all patients that were randomized into a group. Spearman’s rank correlation was used to measure the correlation of BMI with death. A P value was considered statistically significant at the P< 0.05 threshold. This study used SPSS 26.0 for Windows (IBM Corp., Armonk, NY) and GraphPad Prism 9.0 for data processing and statistical analysis.

## Results

### Patients

**Figure 1:**
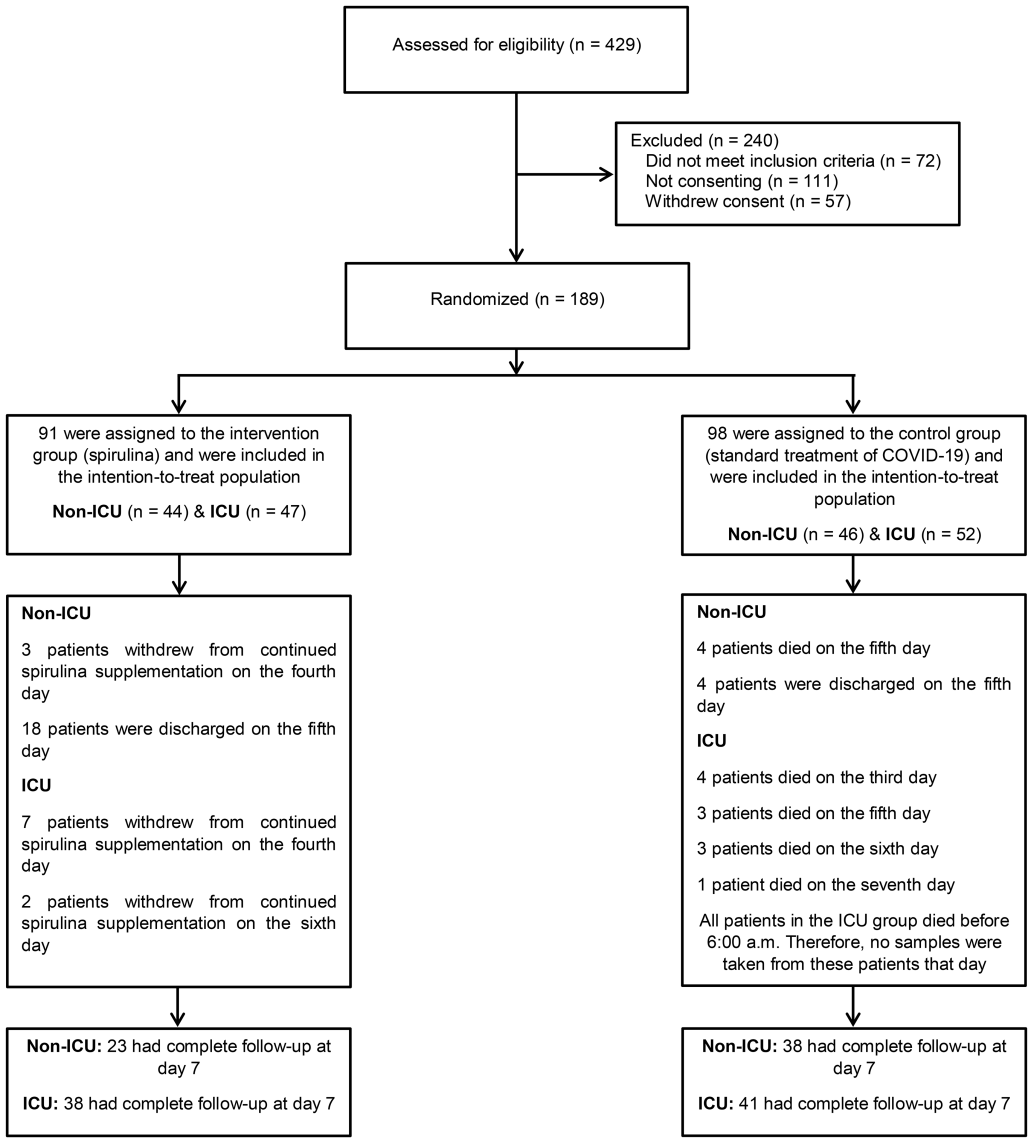
Consort flow chart.

Of the 189 patients who underwent randomization, 44 (mean age = 45.8 y) non-ICU patients (males = 25, 56.8%) and 47 (mean age = 49.4 y) ICU patients (males = 25, 53.2%) were assigned to the *Spirulina* group. There were 46 (mean age = 47.8 y) non-ICU patients (males = 20, 43.5%) and 52 (mean age = 51.5 y) ICU patients (males = 28, 53.8%) assigned to the control group (**Figure 1**). Demographic characteristics and baseline laboratory test results are presented in **Table 1**. There were no essential differences between the *Spirulina* and control groups based on the subcategories of ICU and non-ICU patients.

**Table 1:** Demographic and baseline characteristics.

	Non-ICU		ICU	
	Spirulina (n=44)	Control (n=46)	Spirulina (n=47)	Control (n=52)
Age (years)	45.8 ± 10.9	47.8 ± 10.9	49.4 ± 10.2	51.5 ± 12.6
Male sex, n (%)	25 (56.8)	20 (43.5)	25 (53.2)	28 (53.8)
BMI group, n (%)				
<18.5 kg/m^2^	5 (11.4)	2 (4.3)	3 (6.4)	5 (9.6)
18.5 - 24.9 kg/m^2^	15 (34.1)	13 (28.3)	16 (34.0)	12 (23.1)
25 - 29.9 kg/m^2^	18 (40.9)	24 (52.2)	20 (42.6)	26 (50.0)
30 - 34.9 kg/m^2^	6 (13.6)	7 (15.2)	8 (17.0)	9 (17.3)
Pre-existing conditions, n (%)				
Diabetes	10 (22.7)	16 (34.8)	12 (25.5)	15 (28.8)
Types of Diabetes				
Type 1	2 (20.0)	0 (0.0)	1 (8.3)	3 (20.0)
Type 2	8 (80.0)	16 (100.0)	11 (91.7)	12 (80.0)
Ischemic heart disease	4 (9.1)	5 (10.9)	7 (14.9)	9 (17.3)
Hypertension	13 (29.5)	14 (30.4)	14 (29.8)	19 (36.5)
Smokers	3 (6.8)	5 (10.9)	6 (12.8)	3 (5.8)
Vaccinated	0 (0.0)	2 (4.3)	3 (6.4)	3 (5.8)
First dose – COVID-19 vaccine	–	2 (100.0)	3 (100.0)	2 (66.7)
Second dose – COVID-19 vaccine	–	0 (0.0)	0 (0.0)	1 (33.3)
COVID-19 diagnosis				
RT-PCR (positive), n (%)	34 (77.3)	34 (73.9)	40 (85.1)	45 (86.5)
Re-test (positive), n (%)^a^	10 (100.0)	12 (100.0)	7 (100.0)	7 (100.0)
CT lung involvement percentage categories, n (%)				
0 - 25	1 (2.3)	7 (15.2)	0 (0.0)	0 (0.0)
26 - 50	41 (93.2)	37 (80.5)	43 (91.5)	47 (90.4)
51 - 75	2 (4.5)	2 (4.3)	4 (8.5)	5 (9.6)
76 - 100	0 (0.0)	0 (0.0)	0 (0.0)	0 (0.0)
Clinical manifestation, n (%)				
Sore throat	18 (40.9)	14 (30.4)	15 (31.9)	13 (25.0)
Headache	13 (29.5)	14 (30.4)	14 (29.8)	16 (30.8)
Fatigue	30 (68.2)	19 (41.3)	39 (83.0)	43 (82.7)
Cough	23 (52.3)	15 (32.6)	31 (66.0)	29 (55.8)
Clinical examination				
Temperature (°C), median (IQR)	36.9 (36.8–37.1)	36.9 (36.8–37.2)	36.3 (36.3–36.5)	36.4 (36.3–36.5)
Systolic blood pressure (mmHg), median (IQR)	132.0 (128.5–134.8)	136.0 (131.8–139.3)	141.0 (136.0–145.0)	138.0 (133.0–145.0)
Diastolic blood pressure (mmHg), median (IQR)	83.0 (80.3–84.0)	85.0 (82.8–87.0)	84.0 (81.0–87.0)	82.0 (79.0–86.0)
Oxygen saturation (%) (ICU, with mask), median (IQR)	92.0 (92.0–93.0)	92.0 (92.0–93.0)	90.0 (90.0–90.0)	90.0 (89.0–90.0)
Laboratory values				
AST				
≤39 U/liter, n (%)	43 (97.7)	44 (95.7)	5 (10.6)	4 (7.7)
>39 U/liter, n (%)	1 (2.3)	2 (4.3)	42 (89.4)	48 (92.3)
ALT				
≤36 U/liter, n (%)	43 (97.7)	44 (95.7)	3 (6.4)	7 (13.5)
>36 U/liter, n (%)	1.(2.3)	2 (4.3)	44 (93.6)	45 (86.5)
CRP				
≤35 mg/liter, n (%)	29 (65.9)	36 (78.3)	12 (25.5)	22 (42.3)
>35 mg/liter, n (%)	15 (34.1)	10 (21.7)	35 (74.5)	30 (57.7)
ESR				
≤41 mm/hr, n (%)	31 (70.5)	36 (78.3)	12 (25.5)	18 (34.6)
>41 mm/hr, n (%)	13 (29.5)	10 (21.7)	35 (74.5)	34 (65.4)
Concomitant medications administered as standard of care, n (%)				
Remdesivir	44 (100.0)	46 (100.0)	47 (100.0)	52 (100.0)
Dexamethasone	44 (100.0)	46 (100.0)	47 (100.0)	52 (100.0)
Methylprednisolone	0 (0.0)	4 (8.7)	19 (40.4)	21 (40.4)
Tocilizumab	0 (0.0)	4 (8.7)	2 (4.3)	6 (11.5)
Heparin	44 (100.0)	46 (100.0)	34 (72.3)	35 (67.3)
Enoxaparin	0 (0.0)	8 (17.4)	13 (27.7)	23 (44.2)

Plus-minus values are means ± standard deviation (SD). IQR denotes the interquartile range [median (25^th^ percentile–75^th^ percentile)]. Data were available for 189 patients (in the non-ICU subgroup, 44 patients in the spirulina group, 46 patients in the control group, and in the ICU subgroup, 47 patients in the spirulina group and 52 patients in the control group).

a Immediately after the PCR result was negative, re-sampling and re-testing of the patients were done on the same day. Patients whose re-test results were negative were excluded from the study.

BMI, body mass index; AST, aspartate aminotransferase; ALT, alanine aminotransferase; CRP, C-reactive protein; ESR, erythrocyte sedimentation rate.

The most frequent comorbidities observed in the *Spirulina* group included diabetes (non-ICU = 22.7%; ICU = 25.5%), hypertension (non-ICU = 29.5%; ICU = 29.8%), and ischemic heart disease (non-ICU = 9.1%; ICU = 14.9%). The most frequent comorbidities in the control group included diabetes (non-ICU = 34.8%; ICU = 28.8%), hypertension (non-ICU = 30.4%; ICU = 36.5%), and ischemic heart disease (non-ICU = 10.9%; ICU = 17.3%). During the clinical trial, in addition to the standard treatment of COVID-19, two ICU patients (4.3%) in the *Spirulina* group received tocilizumab, while in the control group, six ICU patients (11.5%) and four non-ICU patients (8.7%) received tocilizumab (**Table 1**).

Moreover, We utilized Spearman’s rank correlation to assess the relationship between BMI and mortality in both ICU and non-ICU control groups. The findings revealed a weak correlation between BMI and mortality in both groups, with correlation coefficients of (*r*=0.30) for the non-ICU group and (*r*=0.28) for the ICU group.

### Clinical laboratory findings and monitoring of immune mediators

In the non-ICU for the *Spirulina* and control groups, the results of clinical laboratory findings on the last follow-up day were as follows: CRP (ARR, 36.84; 95% CI, 21.51 to 52.18, NNT=3; 95% CI, 1.9 to 4.7; *P*<0.001), erythrocyte sedimentation rate (ESR) (ARR, 31.58; 95% CI, 16.80 to 46.36, NNT=4; 95% CI, 2.2 to 6.0; *P* = 0.003), alanine aminotransferase (ALT) (ARR, 28.38; 95% CI, 3.59 to 53.16, NNT=4; 95% CI, 1.9 to 27.8; *P* = 0.03), aspartate aminotransferase (AST) (ARR, 42.33; 95% CI, 19.09 to 65.58, NNT=3; 95% CI, 1.5 to 5.2; *P* = 0.001), lactate dehydrogenase (LDH) (*Spirulina* 660.3 ± 134.8 & control 820.2 ± 136.1; *P*<0.001), and ferritin (*Spirulina*, median 233.6; IQR, 218.4 to 277.2 & control, median 306.4; IQR, 254.9 to 381.5; *P*<0.001) (**Supplementary Table 2**). Likewise, the results of clinical laboratory findings in the ICU for the *Spirulina* and control groups on the last follow-up day were as follows: ESR (ARR, 30.36; 95% CI, 10.71 to 50.01, NNT=4; 95% CI, 2.0 to 9.3; *P* = 0.008), creatinine (*Spirulina*, median 1.1; IQR, 1.1 to 1.3 & control, median 1.3; IQR, 1.2 to 1.4; *P*<0.001), ferritin (*Spirulina*, median 409.7; IQR, 309.1 to 458.0 & control, median 434.1; IQR, 382.9 to 576.0; *P* = 0.02), and D-dimer (*Spirulina*, median 686.5; IQR, 582.0 to 782.5 & control, median 756.0; IQR, 633.5 to 891.0; *P* = 0.03) (**Supplementary Table 2**).

Furthermore, On the last day of follow-up, the results of the monitoring of immune mediators for the *Spirulina* and control groups in non-ICU were as follows: IL-6 (control, median 95.9; IQR, 79.4 to 126.8 & *Spirulina*, median 75.3; IQR, 65.7 to 88.3; *P*<0.001), TNF-α (control, median 47.8; IQR, 37.7 to 60.8 & *Spirulina*, median 35.2; IQR, 28.6 to 46.7; *P* = 0.002), IL-10 (control, median 9.1; IQR, 7.8 to 9.8 & *Spirulina*, median 7.8; IQR, 7.2 to 8.5; *P* = 0.003), IFN-γ (control, median 55.0; IQR, 45.9 to 66.3 & *Spirulina*, median 64.6; IQR, 53.3 to 83.6; *P* = 0.02), IP-10 (control, median 290.7; IQR, 240.9 to 389.8 & *Spirulina*, median 181.5; IQR, 171.5 to 208.7; *P*<0.001) (**Table 2**). Moreover, in ICU, the results of monitoring immune mediators on the last day of follow-up for the *Spirulina* and control groups were as follows: IL-6 (control, median 194.3; IQR, 146.0 to 235.9 & *Spirulina*, median 126.2; IQR, 89.8 to 181.7; *P*<0.001), IFN-γ (control, median 62.8; IQR, 51.6 to 72.3 & *Spirulina*, median 93.5; IQR, 75.0 to 125.9; *P*<0.001), MIP-1α (*Spirulina* 17.4 ± 4.0 & control 20.4 ± 5.0; *P*= 0.005) (**Table 2**).

**Table 2:** Monitoring of immune mediators in the intention-to-treat population.

	Non-ICU		Difference (95% CI)	P Value
	Spirulina (n=44)	Control (n=46)		
Immune mediators				
Pro-inflammatory cytokines				
IL-6 (ng/ml), median (IQR)				
Day 1	90.2 (70.7–146.3)	75.8 (58.2–116.7)	12.9 (-5.5 to 32.0)	0.22
Day 3	92.7 (68.9–121.0)	91.3 (55.6–116.6)	2.4 (-17.2 to 20.0)	0.82
Day 5	70.3 (46.1–107.2)	115.5 (87.7–136.8)	-42.0 (-63.9 to -23.0)	<0.001
Day 7	75.3 (65.7–88.3)	95.9 (79.4–126.8)	-21.5 (-37.9 to -9.4)	<0.001
TNF-α (ng/ml), median (IQR)				
Day 1	51.8 (38.8–68.8)	41.2 (28.1–66.5)	8.1 (-1.8 to 17.9)	0.10
Day 3	44.3 (36.2–61.1)	50.7 (35.2–70.0)	-3.7 (-12.7 to 4.6)	0.46
Day 5	36.5 (29.0–49.4)	46.6 (35.0–64.3)	-10.1 (-18.0 to -3.3)	0.005
Day 7	35.2 (28.6–46.7)	47.8 (37.7–60.8)	-11.8 (-19.0 to -3.8)	0.002
Anti-inflammatory cytokine				
IL-10 (ng/ml), median (IQR)				
Day 1	8.1 (7.1–9.7)	7.7 (6.7–8.9)	0.4 (-0.3 to 1.1)	0.28
Day 3	8.4 (7.3–10.1)	8.6 (7.2–9.5)	0.05 (-0.7 to 0.8)	0.87
Day 5	8.1 (7.4–9.1)	8.9 (7.7–10.6)	-0.9 (-1.6 to -0.1)	0.02
Day 7	7.8 (7.2–8.5)	9.1 (7.8–9.8)	-1.0 (-1.7 to -0.3)	0.003
Inflammatory cytokine				
IFN-γ (ng/ml), median (IQR)				
Day 1	43.1 (37.0–51.7)	45.5 (36.5–55.5)	-3.4 (-8.7 to 2.0)	0.21
Day 3	55.2 (44.2–81.8)	57.8 (47.1–68.3)	-0.07 (-7.8 to 9.3)	0.98
Day 5	77.6 (57.4–95.7)	51.6 (41.3–70.3)	19.8 (10.4 to 31.7)	<0.001
Day 7	64.6 (53.3–83.6)	55.0 (45.9–66.3)	10.0 (1.6 to 19.7)	0.02
Chemokines				
IP-10 (ng/ml), median (IQR)				
Day 1	187.5 (150.7–250.9)	192.1 (167.0–211.6)	1.7 (-19.4 to 26.0)	0.90
Day 3	204.0 (175.9–239.4)	233.8 (201.5–260.2)	-27.0 (-49.2 to -3.9)	0.03
Day 5	198.7 (169.1–227.8)	253.9 (210.6–306.9)	-62.4 (-88.2 to -37.8)	<0.001
Day 7	181.5 (171.5–208.7)	290.7 (240.9–389.8)	-98.4 (-146.6 to -68.5)	<0.001
MIP-1α (ng/ml), median (IQR)				
MIP-1α (day 1), mean (±SD)	23.5 ± 10.2	20.2 ± 6.1	3.3 (-0.2 to 6.9)	0.07
Day 3	22.8 (20.1–27.9)	23.1 (20.7–26.7)	-0.1 (-2.1 to 2.2)	0.92
Day 5	22.9 (19.1–25.7)	25.0 (22.6–28.6)	-2.8 (-5.2 to -0.5)	0.02
Day 7	22.5 (19.9–28.2)	24.7 (20.9–26.8)	-0.3 (-3.4 to 4.3)	0.90
MCP-1 (ng/ml), median (IQR)				
Day 1	195.6 (178.8–223.2)	194.0 (165.1–214.1)	4.8 (-8.5 to 20.4)	0.44
Day 3	209.2 (189.4–238.4)	196.6 (177.8–223.9)	12.0 (-2.4 to 23.8)	0.10
Day 5	202.2 (192.8–220.5)	210.9 (194.1–239.1)	-8.1 (-23.3 to 3.0)	0.15
Day 7	199.7 (187.4–209.0)	207.6 (183.4–228.9)	-6.6 (-20.7 to 5.5)	0.26

	ICU		Difference (95% CI)	P Value
	Spirulina (n=47)	Control (n=52)		
Immune mediators				
Pro-inflammatory cytokines				
IL-6 (ng/ml), median (IQR)				
Day 1	227.6 (119.6–318.6)	226.2 (147.4–321.4)	-6.1 (-55.4 to 39.7)	0.82
IL-6 (day 3), mean (±SD)	197.3 ± 80.1	243.6 ± 100.2	-46.3 (-83.3 to -9.3)	0.01
Day 5	142.2 (105.5–194.2)	211.6 (137.9–287.2)	-62.1 (-102.0 to -27.8)	<0.001
Day 7	126.2 (89.8–181.7)	194.3 (146.0– 235.9)	-61.1 (-93.0 to -32.7)	<0.001
TNF-α (ng/ml), median (IQR)				
Day 1	86.4 (64.8–119.8)	78.2 (62.5–107.5)	7.3 (-5.1 to 21.0)	0.24
TNF-α (day 3), mean (±SD)	87.5 ± 24.0	88.7 ± 29.3	-1.2 (-12.1 to 9.8)	0.83
Day 5	77.4 (60.6–96.1)	77.2 (58.2–95.0)	-1.1 (-13.2 to 11.0)	0.79
Day 7	68.3 (54.1–87.4)	75.4 (58.8–88.2)	-5.1 (-15.9 to 4.6)	0.32
Anti-inflammatory cytokine				
IL-10 (ng/ml), median (IQR)				
IL-10 (day 1), mean (±SD)	13.7 ± 4.9	14.2 ± 3.4	-0.5 (-2.2 to 1.2)	0.59
Day 3	13.9 (11.0–15.7)	14.8 (11.8–17.1)	-0.8 (-2.2 to 0.6)	0.24
Day 5	12.3 (10.8–13.7)	13.5 (11.1–16.2)	-1.2 (-2.8 to 0.2)	0.11
Day 7	11.9 (10.3–13.4)	13.1 (10.8–13.8)	-1.0 (-2.2 to 0.3)	0.12
Inflammatory cytokine				
IFN-γ (ng/ml), median (IQR)				
Day 1	55.4 (39.3–70.6)	57.2 (45.5–77.2)	-6.1 (-16.9 to 3.2)	0.17
Day 3	78.4 (56.8–104.2)	64.0 (47.5–83.0)	13.5 (2.3 to 25.8)	0.02
Day 5	84.7 (69.5–106.4)	67.5 (53.2–84.2)	18.9 (8.1 to 29.5)	0.003
Day 7	93.5 (75.0–125.9)	62.8 (51.6–72.3)	30.8 (19.5 to 42.6)	<0.001
Chemokines				
IP-10 (ng/ml), median (IQR)				
IP-10 (day 1), mean (±SD)	513.1 ± 197.2	554.9 ± 169.0	-41.8 (-114.9 to 31.2)	0.26
Day 3	517.0 (403.6–597.7)	561.5 (434.3–658.5)	-36.5 (-92.1 to 18.9)	0.19
Day 5	450.9 (402.1–542.1)	513.1 (416.1–612.4)	-48.4 (-107.4 to 7.7)	0.10
Day 7	469.8 (411.5–556.7)	483.0 (399.0–547.1)	-2.1 (-53.4 to 49.8)	0.98
MIP-1α (ng/ml), median (IQR)				
MIP-1α (day 1), mean (±SD)	20.4 ± 7.8	21.0 ± 5.4	-0.6 (-3.3 to 2.1)	0.65
Day 3	20.6 (15.8–23.4)	21.9 (17.1–25.8)	-1.2 (-3.4 to 0.9)	0.22
Day 5	19.2 (16.6–21.8)	21.0 (17.2–25.2)	-1.8 (-4.1 to 0.6)	0.15
MIP-1α (day 7), mean (±SD)	17.4 ± 4.0	20.4 ± 5.0	-2.9 (-5.0 to -0.9)	0.005
MCP-1 (ng/ml), median (IQR)				
Day 1	286.6 (229.5–361.8)	303.5 (257.4–343.8)	-15.6 (-45.3 to 17.6)	0.38
Day 3	299.2 (250.4–328.1)	315.2 (264.5–357.1)	-15.8 (-39.5 to 6.0)	0.14
Day 5	272.5 (246.7–299.2)	294.8 (254.1–340.7)	-21.5 (-47.2 to 3.0)	0.10
Day 7	264.5 (238.7–294.4)	286.2 (248.0–300.0)	-17.1 (-37.1 to 3.6)	0.11

Plus-minus values are means ± standard deviation (SD). IQR denotes the interquartile range [median (25^th^ percentile–75^th^ percentile)]. The number of patients (for all variables): in the non-ICU subgroup intervention group (first day n=44, third day n=44, fifth day n=41, and seventh day n=23); control group (first day n=46, third day n=46, fifth day n=46, and seventh day n=38), and the ICU subgroup: intervention group (first day n=47, third day n=47, fifth day n=40, and seventh day n=38); control group (first day n=52, third day n=48, fifth day n=45, and seventh day n=41). Differences were expressed as the median difference (Hodges–Lehmann estimate) or mean difference and 95% confidence intervals.

IL-6, interleukin-6; TNF-α, tumor necrosis factor alpha; IL-10, interleukin-10; IFN-γ, interferon-γ; IP-10, interferon gamma-induced protein 10; MIP-1α, macrophage inflammatory protein 1α; MCP-1, monocyte chemotactic protein 1.

The comparison of immune mediators in groups before and after treatment and between surviving and deceased patients is fully described in **Supplementary Tables 5**, **6**, respectively.

### Primary outcome

**Figure 2:**
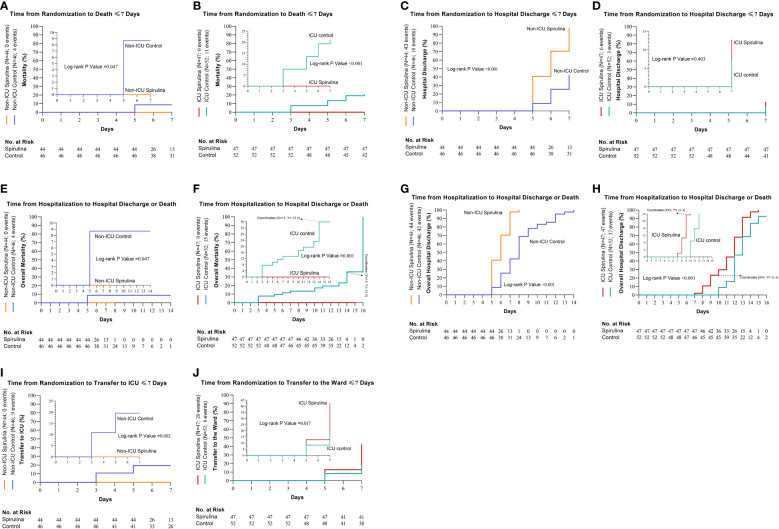
Cumulative incidence of the primary and secondary outcomes. The primary outcomes were death and hospital discharge ≤7 days (Day Since Randomization) (death; non-ICU **(A)** and ICU **(B)**, hospital discharge; non-ICU **(C)** and ICU **(D)**. The secondary outcomes were overall deaths, overall hospital discharge, and also transfer between hospital wards ≤7 days (overall deaths; non-ICU **(E)** and ICU **(F)**, overall hospital discharge; non-ICU **(G)** and ICU **(H)**, transfer to ICU for the non-ICU subgroup, **(I)** and transfer to the ward for ICU subgroup, **(J)**).

By the seventh day of follow-up, four (8.7%) out of 46 non-ICU patients in the control group died compared to no deaths in the *Spirulina* group (HR, 0.13; 95% CI, 0.02 to 0.97; *P* = 0.047) (**Figure 2A**). Likewise, by the seventh-day follow-up, 11 (21.2%) out of 52 ICU patients in the control group died compared to no deaths in the *Spirulina* group (HR, 0.13; 95% CI, 0.04 to 0.44; *P*<0.001) (**Figure 2B**). There were 43 (97.7%) out of 44 non-ICU patients in the *Spirulina* group discharged compared to 18 (39.1%) out of 46 non-ICU patients in the control group after seven days (HR, 6.52; 95% CI, 3.50 to 12.17; *P*<0.001) (**Figure 2C**). Additionally, a greater percentage of ICU patients in the *Spirulina* group (n=6, 12.8%) were discharged compared to the control group (n=3, 5.8%) (HR, 1.80; 95% CI, 0.46 to 7.11; *P* = 0.403) (**Table 3**) (**Figure 2D**).

**Table 3:** Outcomes in the intention-to-treat population.

	Non-ICU		Difference (95% CI)	NNT or NNH (95% CI)	P Value
	Spirulina (n=44)	Control (n=46)			
Primary outcome					
Death ≤7 days (Days Since Randomization), n (%)	0 (0.0)	4 (8.7)	HR 0.13 (0.02 to 0.97)		0.047
			ARR 8.70 (0.55 to 16.84)	NNT=12 (5.9 to 180.8)	
Hospital Discharge ≤7 days (Days Since Randomization), n (%)	43 (97.7)	18 (39.1)	HR 6.52 (3.50 to 12.17)		<0.001
			ARR 58.60 (43.82 to 73.37)	NNT=2 (1.4 to 2.3)	
Secondary outcomes					
Overall deaths, n (%)	0 (0.0)	4 (8.7)	HR 0.13 (0.02 to 0.97)		0.047
			ARR 8.70 (0.55 to 16.84)	NNT=12 (5.9 to 180.8)	
Overall Hospital Discharge, n (%)	44 (100.0)	42 (91.3)	HR 6.56 (3.54 to 12.14)		<0.001
			ARR 8.70 (0.55 to 16.84)	NNT=12 (5.9 to 180.8)	
Transfer to ICU, n (%)	0 (0.0)	9 (19.6)	HR 0.12 (0.03 to 0.47)		0.002
			ARR 19.57 (8.10 to 31.03)	NNT=6 (3.2 to 12.3)	
Hospitalization (days), median (IQR)	6.0 (5.0–7.0)	8.0 (6.0–9.0)	-2.0 (-2.0 to -1.0)		<0.001
ICU length of stay (days), median (IQR)	–	5.0 (2.0–7.0)	–		
Subsidiary clinical outcomes					
Clinical manifestation^a^					
Sore throat, n (%)					
Day 1	18 (40.9)	14 (30.4)	–	–	0.30
Day 3	5 (11.4)	14 (32.6)	ARR 21.19 (4.34 to 38.05)	NNT=5 (2.6 to 23.0)	0.02
Day 5	2 (4.9)	13 (31.0)	ARR 26.07 (10.62 to 41.53)	NNT=4 (2.4 to 9.4)	0.002
Day 7	0 (0.0)	11 (28.9)	ARR 28.95 (14.53 to 43.37)	NNT=4 (2.3 to 6.9)	0.004
Headache, n (%)					
Day 1	13 (29.5)	14 (30.4)	–	–	0.93
Day 3	4 (9.1)	12 (27.9)	ARR 18.82 (2.95 to 34.69)	NNT=6 (2.9 to 34.0)	0.02
Day 5	5 (12.2)	10 (23.8)	ARR 11.61 (-4.70 to 27.93)	NNT=9 (Helpful >3.6) (Harmful >21.3)	0.17
Day 7	5 (21.7)	9 (23.7)	ARR 1.95 (-19.66 to 23.55)	NNT=52 (Helpful >4.2) (Harmful >5.1)	0.86
Fatigue, n (%)					
Day 1	30 (68.2)	19 (41.3)	–	–	0.01
Day 3	16 (36.4)	23 (53.5)	ARR 17.12 (-3.47 to 37.72)	NNT=6 (Helpful >2.7) (Harmful >28.8)	0.11
Day 5	12 (29.3)	24 (57.1)	ARR 27.87 (7.43 to 48.32)	NNT=4 (2.1 to 13.5)	0.01
Day 7	7 (30.4)	21 (55.3)	ARR 24.83 (0.26 to 49.40)	NNT=5 (2.0 to 382.8)	0.06
Cough, n (%)					
Day 1	23 (52.3)	15 (32.6)	–	–	0.06
Day 3	11 (25.0)	13 (30.2)	ARR 5.23 (-13.53 to 24.00)	NNT=20 (Helpful >4.2) (Harmful >7.4)	0.59
Day 5	5 (12.2)	10 (23.8)	ARR 11.61 (-4.70 to 27.93)	NNT=9 (Helpful >3.6) (Harmful >21.3)	0.17
Day 7	2 (8.7)	10 (26.3)	ARR 17.62 (-0.51 to 35.75)	NNT=6 (Helpful >2.8) (Harmful >196.9)	0.09
Clinical examination^b^					
Temperature (°C), median (IQR)					
Day 1	36.9 (36.8–37.1)	36.9 (36.8–37.2)	0 (-0.1 to 0)		0.35
Day 3	36.6 (36.5–36.7)	36.5 (36.5–36.6)	0 (0 to 0.1)		0.29
Day 5	36.6 (36.6–36.8)	36.6 (36.5–36.7)	0.1 (0 to 0.1)		0.03
Day 7	36.8 (36.7–36.9)	36.6 (36.5–36.7)	0.2 (0.1 to 0.3)		<0.001
Oxygen saturation (%), median (IQR)					
Day 1	92.0 (92.0–93.0)	92.0 (92.0–93.0)	0 (0 to 0)		0.77
Day 3	93.0 (93.0–94.0)	92.0 (92.0–92.3)	1.0 (1.0 to 2.0)		<0.001
Day 5	95.0 (94.0–95.0)	92.0 (92.0–93.0)	3.0 (2.0 to 3.0)		<0.001
Day 7	95.0 (95.0–96.0)	92.0 (92.0–93.0)	3.0 (3.0 to 4.0)		<0.001

	ICU		Difference (95% CI)	NNT or NNH (95% CI)	P Value
	Spirulina (n=47)	Control (n=52)			
Primary outcome					
Death ≤7 days (Days Since Randomization), n (%)	0 (0.0)	11 (21.2)	HR 0.13 (0.04 to 0.44)		<0.001
			ARR 21.15 (10.05 to 32.25)	NNT=5 (3.1 to 9.9)	
Hospital Discharge ≤7 days (Days Since Randomization), n (%)	6 (12.8)	3 (5.8)	HR 1.80 (0.46 to 7.11)		0.403
			ARR 7.00 (-4.46 to 18.45)	NNT=15 (Helpful >5.4) (Harmful >22.4)	
Secondary outcomes					
Overall deaths, n (%)	0 (0.0)	15 (28.8)	HR 0.16 (0.05 to 0.48)		0.001
			ARR 28.85 (16.53 to 41.16)	NNT=4 (2.4 to 6.0)	
Overall Hospital Discharge, n (%)	47 (100.0)	37 (71.2)	HR 2.59 (1.52 to 4.44)		<0.001
			ARR 28.85 (16.53 to 41.16)	NNT=4 (2.4 to 6.0)	
Transfer to the Ward, n (%)	20 (42.6)	8 (15.4)	HR 2.69 (1.19 to 6.08)		0.017
			ARR 27.17 (9.96 to 44.37)	NNT=4 (2.3 to 10.0)	
Hospitalization (days), median (IQR)	12.0 (10.0–13.0)	12.0 (10.3–13.0)	-1.0 (-1.0 to 0)		0.19
ICU length of stay (days), median (IQR)	8.0 (7.0–9.0)	8.0 (6.0–9.0)	0 (-1.0 to 1.0)		0.84
Subsidiary clinical outcomes					
Clinical manifestation^a^					
Sore throat, n (%)					
Day 1	15 (31.9)	13 (25.0)	–	–	0.45
Day 3	5 (10.6)	12 (26.7)	ARR 16.03 (0.39 to 31.67)	NNT=7 (3.2 to 258.1)	0.048
Day 5	3 (7.5)	7 (16.7)	ARR 9.17 (-4.75 to 23.08)	NNT=11 (Helpful >4.3) (Harmful >21.1)	0.20
Day 7	2 (5.3)	7 (17.1)	ARR 11.81 (-1.72 to 25.34)	NNT=9 (Helpful >3.9) (Harmful >58.1)	0.16
Headache, n (%)					
Day 1	14 (29.8)	16 (30.8)	–	–	0.92
Day 3	6 (12.8)	11 (24.4)	ARR 11.68 (-4.09 to 27.45)	NNT=9 (Helpful >3.6) (Harmful >24.4)	0.15
Day 5	5 (12.5)	13 (31.0)	ARR 18.45 (1.12 to 35.79)	NNT=6 (2.8 to 89.5)	0.04
Day 7	4 (10.5)	10 (24.4)	ARR 13.86 (-2.51 to 30.23)	NNT=8 (Helpful >3.3) (Harmful >39.9)	0.11
Fatigue, n (%)					
Day 1	39 (83.0)	43 (82.7)	–	–	0.97
Day 3	16 (34.0)	40 (88.9)	ARR 54.85 (38.48 to 71.21)	NNT=2 (1.4 to 2.6)	<0.001
Day 5	18 (45.0)	32 (76.2)	ARR 31.19 (11.10 to 51.28)	NNT=4 (2.0 to 9.0)	0.004
Day 7	11 (28.9)	31 (75.6)	ARR 46.66 (27.15 to 66.17)	NNT=3 (1.5 to 3.7)	<0.001
Cough, n (%)					
Day 1	31 (66.0)	29 (55.8)	–	–	0.30
Day 3	18 (38.3)	21 (46.7)	ARR 8.37 (-11.77 to 28.51)	NNT=12 (Helpful >3.5) (Harmful >8.5)	0.42
Day 5	10 (25.0)	14 (33.3)	ARR 8.33 (-11.25 to 27.91)	NNT=12 (Helpful >3.6) (Harmful >8.9)	0.41
Day 7	3 (7.9)	11 (26.8)	ARR 18.93 (2.89 to 34.98)	NNT=6 (2.9 to 34.6)	0.04
Clinical examination^b^					
Temperature (°C), median (IQR)					
Day 1	36.3 (36.3–36.5)	36.4 (36.3–36.5)	-0.05 (-0.1 to 0)		0.12
Day 3	36.4 (36.4–36.5)	36.4 (36.3–36.5)	0 (0 to 0.1)		0.47
Day 5	36.5 (36.4–36.6)	36.5 (36.3–36.6)	0.1 (0 to 0.1)		0.08
Day 7	36.5 (36.4–36.6)	36.5 (36.4–36.6)	0 (-0.1 to 0.1)		0.92
Oxygen saturation (%) (ICU, with mask), median (IQR)					
Day 1	90.0 (90.0–90.0)	90.0 (89.0–90.0)	0 (0 to 0)		0.10
Day 3	91.0 (91.0–91.0)	90.0 (89.0–90.0)	1.0 (1.0 to 1.0)		<0.001
Day 5	92.0 (91.0–93.0)	90.0 (89.0–90.0)	2.0 (2.0 to 3.0)		<0.001
Day 7	92.0 (91.0–94.3)	90.0 (89.0–90.0)	2.0 (2.0 to 4.0)		<0.001

IQR denotes the interquartile range [median (25^th^ percentile–75^th^ percentile)]. Differences were expressed as the median difference (Hodges–Lehmann estimate), or number needed to treat (NNT) with absolute risk reduction (ARR), the number needed to harm (NNH) with absolute risk increase (ARI), and 95% confidence intervals. Hazard ratios (HR, Mantel-Haenszel) were estimated for non-numerical variables of primary and secondary outcomes; the P Value for this ratio was calculated with a log-rank (Mantel-Cox) test.

a The number of patients (Clinical manifestation): in the non-ICU subgroup intervention group (first day n=44, third day n=44, fifth day n=41, and seventh day n=23); control group (first day n=46, third day n=43, fifth day n=42, and seventh day n=38), and the ICU subgroup: intervention group (first day n=47, third day n=47, fifth day n=40, and seventh day n=38); control group (first day n=52, third day n=45, fifth day n=42, and seventh day n=41).

b The number of patients (Clinical examination): in the non-ICU subgroup intervention group (first day n=44, third day n=44, fifth day n=41, and seventh day n=23); control group (first day n=46, third day n=46, fifth day n=46, and seventh day n=38), and the ICU subgroup: intervention group (first day n=47, third day n=47, fifth day n=40, and seventh day n=38); control group (first day n=52, third day n=48, fifth day n=45, and seventh day n=41).

### Secondary outcomes

There were deaths in non-ICU patients for the control group (n=4), but none were found in the *Spirulina* group (HR, 0.13; 95% CI, 0.02 to 0.97; *P* = 0.047) (**Figure 2E**). In the ICU subgroup, 15 out of 52 patients died in the control group, while no deaths occurred in the *Spirulina* group (HR, 0.16; 95% CI, 0.05 to 0.48; *P* = 0.001) (**Figure 2F**). In non-ICU, the overall hospital discharge was (n=42) for the control group and (n=44) for the *Spirulina* group (HR, 6.56; 95% CI, 3.54 to 12.14; *P*<0.001) (**Figure 2G**). Overall hospital discharge in ICU was (n=37) for the control group and (n=47) for the *Spirulina* group (HR, 2.59; 95% CI, 1.52 to 4.44; *P*<0.001) (**Figure 2H**). Nine non-ICU patients in the control group were transferred to ICU, while no such occurrences resulted for the *Spirulina* group (HR, 0.12; 95% CI, 0.03 to 0.47; *P* = 0.002) (**Figure 2I**). During the seven days of follow-up in the ICU subgroup, 20 patients in the *Spirulina* group were transferred to Ward; by contrast, eight patients were transferred to Ward in the control group (HR, 2.69; 95% CI, 1.19 to 6.08; *P* = 0.017) (**Table 3**) (**Figure 2J**).

The median days of hospitalization in the non-ICU subgroup in the control group were eight days (IQR, 6.0 to 9.0), compared to six days in the *Spirulina* group (IQR, 5.0 to 7.0). Furthermore, in the ICU subgroup, the median days of hospitalization in the *Spirulina* and control groups were 12 days (*Spirulina*, IQR, 10.0 to 13.0; control, IQR, 10.3 to 13.0). The median stay in the ICU in the non-ICU subgroup in the control group was five days (for 9 out of 46 patients transferred to the ICU) (IQR, 2.0 to 7.0). Also, in the ICU subgroup, the median stay in the ICU was eight days in the *Spirulina* and control groups (*Spirulina*, IQR, 7.0 to 9.0; control, IQR, 6.0 to 9.0) (**Table 3**).

### Safety

No side effects related to the use of *Spirulina* supplements were observed in the patients during the seven days of follow-up. Some patients were dissatisfied with the high number of daily *Spirulina* supplement capsules taken (i.e., 19 capsules). Eventually, withdrawals from the study due to the difficulty of taking the capsules occurred on the fourth day for three non-ICU patients and seven ICU patients and on the sixth day for two patients in the ICU. One of the notable points during this study was that patients were more willing to take *Spirulina* supplement tablets or powder than the capsule form of this supplement. Patients expressed that if this supplement were in tablet or powder form with liquids or soup, it would be easier to consume, which should be considered in future studies.

## Discussion

The findings of this study indicate that the microalga *Spirulina platensis* could be a beneficial dietary supplement in treating COVID-19. To date, only 30 clinical trials (based on PubMed search results) have been published on *Spirulina platensis* supplementation. To the best of our knowledge, this study represents the first published report of a clinical trial examining high-dose *Spirulina platensis* as a dietary supplement in hospitalized COVID-19 patients. The majority of previous investigations have been centered on animal and *in vitro* studies. The collective evidence has affirmed the antioxidant, anti-inflammatory, antiviral, and antimicrobial properties of this microalga, underscoring its potential benefits as a dietary supplement for human health (20, 21, 30, 34, 40, 41).

The overall results of the present study showed that a six-day course of *Spirulina platensis* plus the standard treatment of COVID-19 was superior in the recovery of patients compared to standard treatment alone. There were no deaths in the *Spirulina* group in contrast to the control group, where four deaths in the non-ICU subgroup and 11 deaths in the ICU subgroup during seven days of follow-up occurred. Patients who received *Spirulina* supplementation in the non-ICU subgroup had a shorter recovery time than those who received only standard COVID-19 treatment, with 97.7% of these patients discharged in ≤7 days. However, there was no significant difference in discharge in the ICU subgroup between the patients who received *Spirulina platensis* and the control group during the seven days of follow-up.

Overall, death in the control group was four patients in non-ICU and 15 in the ICU; on the other hand, there was no death in the *Spirulina* group. During the seven days of follow-up in the non-ICU subgroup, none of the patients who received *Spirulina* supplements were transferred to the ICU, while nine patients in the control group were transferred to the ICU. Additionally, in the ICU subgroup, patients receiving *Spirulina* supplements were transferred from the ICU to the Ward earlier than the control group within seven days. The median hospital stay in the non-ICU patients receiving *Spirulina* was reduced by two days compared to the control group (*Spirulina*, 6; IQR, 5 to 7; control, 8; IQR, 6 to 9). However, there was no significant difference between groups for ICU patients in being hospitalized and staying in the ICU.

Our study focused on analyzing in-hospital mortality rates, and the control group’s death rate (19.4%) aligns with the CDC’s (Centers for Disease Control and Prevention) reported range (10-20%) for adults aged ≥65 years from June 2021 to February 2022 (42).

*Spirulina*, a superfood rich in nutrients, boasts a variety of vitamins, minerals, and beneficial compounds, making it a potential dietary supplement for COVID-19 patients. It is particularly renowned for its high protein content, vitamins A, C, D, E, and K, and minerals such as Calcium, Iron, Selenium, Copper, Zinc, and Magnesium (20). The majority of studies attribute *Spirulina’s* anti-inflammatory, antioxidant, and antiviral properties primarily to two effective substances, Ca-Sp and PCB (20–22). Ca-Sp, a sulfated polysaccharide, has been shown in *in vitro* studies to inhibit the proliferation of various enveloped viruses, including mumps, measles, CMV, IAV, HSV-1, and HIV-1 (28–31). Additionally, *Spirulina platensis* is rich in pigments; phycobili proteins, an important group of auxiliary photosynthetic pigments in cyanobacteria, play a crucial role in light absorption. C-phycocyanin (C-PC), one of the most significant biliproteins of *Spirulina platensis*, has been found to exhibit anti-radical and antioxidant activities. Studies have also underscored its potential anti-cancer effects, which include blocking cell cycle progression, inducing autophagy and apoptosis in cancer cells, and acting as a selective cyclooxygenase 2 (COX-2) inhibitor (20–22, 34, 43). Notably, a clinical trial involving naïve HIV-1 patients in Cameroon demonstrated that patients who received *Spirulina* experienced a significant reduction in viral load and, conversely, an increase in CD4 cells (17). Given this microalga’s antiviral, anti-inflammatory, and antioxidant properties, and considering the absence of fatalities and shorter hospitalization time in the intervention group, it appears that *Spirulina*, in conjunction with standard COVID-19 treatment, may have contributed to the improved recovery of COVID-19 patients by engaging key pathways.

The highest *Spirulina* dose was used in a clinical trial on HIV-1 patients who received 19 g of *Spirulina platensis* daily (17). Our study chose the daily dose of 15.2 g of *Spirulina* because, On average, each 15 g of *Spirulina* provides about 100 mg of PCB, which exerts a wide range of anti-inflammatory and antioxidant effects (22, 34).

The oxygen saturation level in patients who received *Spirulina* increased significantly compared to patients who received the standard treatment of COVID-19 alone. In non-ICU patients, the median oxygen saturation in the *Spirulina* group reached 95% on the fifth and seventh days of follow-up. In comparison, the oxygen saturation in the control group reached only 92%. Furthermore, in ICU patients, the median oxygen saturation of those who received *Spirulina* reached 92% on the fifth and seventh days of follow-up. By contrast, the oxygen saturation was only 90% in the patients who received the standard treatment of COVID-19 alone. Moreover, the clinical laboratory findings in the non-ICU subgroup showed that in patients receiving *Spirulina* compared to the control group, there was a decrease in CRP, ESR, ALT, AST, LDH, and ferritin levels as the intervention duration increased. Likewise, ICU patients receiving *Spirulina* supplements compared to the control group showed significant reductions in ESR, creatinine, ferritin, and D-dimer with increasing intervention time.

In severe cases of COVID-19, an excessive immune response leads to multiple organ dysfunctions due to the uncontrolled production of inflammatory mediators (9, 10). Macrophage activation causes this severe immune response, increasing cytokine production such as TNF-α and IL-6 and biomarkers such as CRP, ferritin, and IP-10. Excessive inflammation aggravates ARDS, which is one of the primary causes of death in COVID-19 patients (9, 10, 12, 14, 15). The IL-6 and TNF-α cytokines play a crucial role in inducing an inflammatory state (9, 10). Studies show that a positive correlation exists between IL-6 and CRP levels in COVID-19 patients (10, 11, 15). The increase in CRP levels predicts respiratory deterioration, and a significant increase in baseline IL-6 levels is positively associated with the progression of pulmonary involvement (11, 15). However, a rapid and sustained decrease in CRP levels was reported in COVID-19 patients receiving tocilizumab, which is an IL-6 receptor monoclonal antibody (15). Overall, particularly in the intervention group, reducing IL-6 levels seems to have a positive effect on reducing inflammatory biomarkers and improving oxygen saturation status.

The monitoring of immune mediators in non-ICU patients showed that patients who received *Spirulina* compared to the control group significantly decreased their levels of IL-6, TNF-α, IL-10, and IP-10 as the intervention time increased. On the other hand, in non-ICU patients, the level of IFN-γ in the *Spirulina* group was significantly higher than in the control group. Moreover, there was no significant difference in MCP-1 and MIP-1α levels between the patients who received *Spirulina* supplementation compared to the control group during seven days of follow-up. Likewise, the levels of MIP-1α and IL-6 in the ICU patients who received *Spirulina* supplements decreased significantly as the intervention progressed. Like the non-ICU subgroup, in the ICU subgroup, the level of IFN-γ in the intervention group was significantly higher than in the control group. Also, no significant difference was observed in the ICU in TNF-α, IL-10, IP-10, and MCP-1 levels between the *Spirulina* and control groups.

Several studies suggest that macrophages are responsible for the cytokine storm seen in severe COVID-19 patients, but it is unclear how this occurs since macrophages are not directly infected by SARS-CoV-2 (9, 10, 14). One study found that pDCs, which are directly infected with SARS-CoV-2 and trigger TLR-7 activation, are the main producers of IFN-I during disease. Subsequently, IFN-I produced due to the infection of pDCs by SARS-CoV-2 causes a macrophage-mediated cytokine storm in COVID-19 patients (14). The noncanonical NF-κB pathway controls the production of IFN-I in antiviral innate immunity (32). Activating the transcription factor NF-κB in various cells, including macrophages in the lungs, causes the production of pro-inflammatory cytokines such as IL-6 and TNF-α (32, 33, 44). Studies indicate that inhibiting the NF-κB pathway has a potential therapeutic role in COVID-19 patients (10, 40). *Spirulina* has been shown to inhibit the NF-κB pathway, induce Nrf2 activation, and may have therapeutic potential in treating COVID-19 (34–39).

Evidence shows that the composition of the gut and lung microbiota in COVID-19 patients changes; specifically, these changes in the gut microbiota show that the abundance of beneficial bacteria such as *Faecalibacterium prausnitzii*, which is one of the producers of short-chain fatty acids (SCFAs), has decreased (9, 45). Studies show that the gut microbiota has key effects on the metabolic processes of the host as well as the development and regulation of the body’s immune system. Intestinal microbes coordinate immune homeostasis by producing metabolites such as butyrate, acetate, and propionate, and these SCFAs induce pro-inflammatory responses and cytokines (9, 46, 47). Considering that the evidence shows that SARS-CoV-2 causes dysbiosis of the gut microbiota and ultimately leads to disruption of the gut-lung axis, many studies suggest using probiotics and prebiotics as adjunctive treatment in COVID-19 patients (48, 49). Microalgae, especially *Spirulina*, are an important source of prebiotics (50). Previous studies confirm that *Spirulina* can modulate gut microbiota composition; these studies show that *Spirulina* consumption increases *Roseburia* and *Lactobacillus*, which may be associated with improved health status (34, 51, 52). Considering the rapid increase in oxygen saturation levels and significant changes in the levels of immune mediators in the intervention group, *Spirulina* plus standard treatment of COVID-19 may improve recovery by involving the NF-κB pathway and modulating the gut-lung axis.

Generally, the monitoring of immune mediators showed that consumption of *Spirulina* supplement causes significant changes in the levels of immune mediators, which, due to the absence of death and shorter hospitalization time of patients in the intervention group, it can be concluded that receiving this microalga, it has brought positive results in COVID-19 patients. Also, *Spirulina* consumption increased the IFN-γ level in the intervention group compared to those in the control group. Studies show that increasing the level of IFN-γ stimulates the immune system and increases the body’s resistance to the invasion of pathogens (10, 53). One of the most important results in this clinical trial was a significant decrease in IL-6 levels, as opposed to a significant increase in IFN-γ levels in the intervention group. IL-6 is one of the complex cytokines because it is produced by non-immune and immune cells across multiple organ systems and affects them. After all, high IL-6 levels are strongly associated with disease progression and shorter survival (9–11). Controlling the cytokine storm and thus reducing the inflammation caused by COVID-19 is one of the main treatment approaches in dealing with this disease (10). Nevertheless, in this study, due to the reduction of immune mediators levels such as IL-6 and the simultaneous increase of IFN-γ in the intervention group, it cannot be concluded that *Spirulina* has controlled the cytokine storm. However, it can be concluded that these changes in immune mediators in patients receiving *Spirulina* have brought positive results, and understanding and describing the mechanism of these positive changes requires innovative studies.

There are limitations to this study that should be acknowledged when interpreting the findings. First of all, this clinical trial was not blinded. Initially, this study was a single-blind trial; nonetheless, Iranian patients negatively viewed the placebo, and none of the patients participated in the study, leading to the change of the study method from a single-blind trial to an open-label trial. Moreover, one of the limitations of this study, as well as other ongoing studies in Iran, is that the data obtained from the follow-up of patients after discharge from the hospital is unreliable because the use of traditional and herbal medicines is widespread among the people of Iran. Furthermore, traditional and herbal medicine use increased significantly during the COVID-19 pandemic (54, 55). Even though follow-up of patients after discharge from the hospital was not among the objectives of this study, we tried our best to follow up with patients long-term. However, patients were not interested in follow-up after discharge. The main reasons for patients’ non-cooperation for follow-up after discharge from the hospital were the fear of contracting the disease again, the unpleasant feeling towards the hospital environment, and the busy work.

## Conclusions

This study showed that a high dose of *Spirulina* combined with standard treatment of COVID-19 significantly accelerates clinical recovery and reduces mortality to zero. Clinical laboratory findings confirm the effective role of this microalga in reducing inflammatory biomarkers such as CRP, ESR, and ferritin. Moreover, the results of monitoring immune mediators in patients who received *Spirulina* supplements show that the levels of IL-6, which is a pro-inflammatory cytokine, decrease significantly. On the other hand, the levels of IFN-γ, which has antiviral activity and important immunoregulatory functions, significantly increase. Considering the reduction of hospitalization time and the absence of death in the intervention group, the results of changes in immune mediators in patients receiving *Spirulina* supplements can be evaluated as beneficial. Presently, it is unclear whether this microalga helps the recovery of COVID-19 patients due to balancing the immune system and reducing inflammation or via other vital pathways. However, these preliminary data will undoubtedly help future studies further investigate this dietary supplement and other drugs in treating COVID-19. Also, the promising findings of this study, considering the availability and affordability of this dietary supplement, show a clear perspective of the potential of this microalga in reducing the hospital burden, especially in the ICU. Moreover, it is suggested that studies with larger sample sizes, long-term follow-up, and adding *Spirulina* supplements to other treatment approaches for COVID-19 should be conducted.

## Data availability statement

The raw data supporting the conclusions of this article will be made available by the authors, without undue reservation.

## Ethics statement

This study is a multicenter, randomized controlled trialapproved by the Research Ethics Committee of Tehran University of Medical Sciences (IR.TUMS.IKHC.REC.1399.481) and the Iranian Registry of Clinical Trials (IRCT) registry team. The study protocol is registered with IRCT under IRCT20210216050373N1, available at https://irct.ir/trial/54375. All patients gave written informed consent before starting the study.

## Author contributions

MA: Conceptualization, Data curation, Formal analysis, Investigation, Methodology, Project administration, Software, Supervision, Validation, Visualization, Writing – original draft, Writing – review & editing. MB: Conceptualization, Data curation, Formal analysis, Investigation, Methodology, Project administration, Software, Supervision, Validation, Visualization, Writing – original draft, Writing – review & editing. SJM: Conceptualization, Data curation, Investigation, Methodology, Project administration, Supervision, Validation, Visualization, Writing – review & editing. HH: Methodology, Project administration, Supervision, Validation, Visualization, Writing – review & editing, Conceptualization, Data curation, Investigation. AM: Conceptualization, Data curation, Investigation, Methodology, Project administration, Supervision, Validation, Visualization, Writing – original draft, Writing – review & editing. MJ-S: Conceptualization, Data curation, Formal analysis, Investigation, Methodology, Software, Validation, Visualization, Writing – original draft, Writing – review & editing. NoS: Data curation, Investigation, Methodology, Project administration, Supervision, Writing – review & editing. PR: Conceptualization, Data curation, Investigation, Methodology, Supervision, Validation, Visualization, Writing – original draft, Writing – review & editing. RT: Conceptualization, Data curation, Formal analysis, Investigation, Methodology, Software, Supervision, Validation, Visualization, Writing – original draft, Writing – review & editing. MH-F: Conceptualization, Data curation, Investigation, Methodology, Supervision, Validation, Visualization, Writing – original draft, Writing – review & editing. GB: Conceptualization, Data curation, Investigation, Methodology, Supervision, Validation, Visualization, Writing – original draft, Writing – review & editing. ZP: Data curation, Investigation, Supervision, Validation, Visualization, Writing – review & editing. SH: Data curation, Investigation, Supervision, Writing – review & editing. GM: Data curation, Investigation, Supervision, Writing – review & editing. RK: Data curation, Investigation, Supervision, Writing – review & editing. SR: Data curation, Investigation, Supervision, Writing – review & editing. SK: Data curation, Investigation, Supervision, Writing – review & editing. MK: Data curation, Investigation, Supervision, Writing – review & editing. GG: Data curation, Investigation, Supervision, Writing – review & editing. NiS: Data curation, Investigation, Supervision, Writing – review & editing. MI: Data curation, Investigation, Supervision, Writing – review & editing. AZ: Data curation, Investigation, Methodology, Project administration, Supervision, Writing – review & editing. SJ: Conceptualization, Formal analysis, Investigation, Methodology, Software, Validation, Visualization, Writing – original draft, Writing – review & editing. OD: Conceptualization, Formal analysis, Investigation, Methodology, Software, Validation, Visualization, Writing – review & editing. SS: Conceptualization, Data curation, Formal analysis, Investigation, Methodology, Project administration, Software, Supervision, Validation, Visualization, Writing – original draft, Writing – review & editing. DH: Conceptualization, Formal analysis, Investigation, Methodology, Software, Validation, Visualization, Writing – original draft, Writing – review & editing.
