# Supplementary material for: Effect of high-dose Spirulina supplementation on hospitalized adults with COVID-19: a randomized controlled trial
Source: Front Immunol. 2024 Apr 8;15:1332425. doi: 10.3389/fimmu.2024.1332425 (PMC11036872; doi:10.3389/fimmu.2024.1332425)
Supplement: Supplementary file 1 [file Table_2.docx]

| TABLE 2 Clinical laboratory findings in the intention-to-treat population. | | | | | | | | | | | | |
| --- | --- | --- | --- | --- | --- | --- | --- | --- | --- | --- | --- | --- |
|  | **Non-ICU** | | | | |  | **Difference (95% CI)** | |  | **NNT or NNH (95% CI)** |  | **P Value** |
|  | **Spirulina (n=44)** | |  | **Control (n=46)** | |  |  | |  |  |  |  |
| Creatinine (mg/dl), median (IQR) |  |  |  |  |  |  |  |  |  |  |  |  |
| Day 1 | 1.2 (1.0–1.2) | |  | 1.1 (0.96–1.2) | |  | 0.04 (-0.01 to 0.13) | |  |  |  | 0.17 |
| Day 3 | 1.1 (1.0–1.2) | |  | 1.2 (0.94–1.3) | |  | 0 (-0.08 to 0.07) | |  |  |  | 0.97 |
| Day 5 | 1.2 (0.98–1.2) | |  | 1.2 (0.96–1.4) | |  | -0.05 (-0.16 to 0.02) | |  |  |  | 0.19 |
| Day 7 | 1.2 (1.1–1.3) | |  | 1.2 (0.97–1.3) | |  | -0.02 (-0.11 to 0.07) | |  |  |  | 0.68 |
| D-dimer (ng/ml), median (IQR) |  |  |  |  |  |  |  |  |  |  |  |  |
| Day 1 | 459.5 (388.3–560.0) | |  | 428.0 (395.3–496.0) | |  | 30.0 (-19.0 to 72.0) | |  |  |  | 0.22 |
| Day 3 | 516.5 (466.0–612.0) | |  | 478.0 (417.0–539.0) | |  | 43.0 (6.0 to 83.0) | |  |  |  | 0.03 |
| Day 5 | 566.0 (507.5–623.0) | |  | 512.5 (476.8–613.3) | |  | 31.0 (-14.0 to 71.0) | |  |  |  | 0.14 |
| Day 7 | 585.0 (546.0–638.0) | |  | 574.0 (520.3–632.3) | |  | 23.5 (-16.0 to 64.0) | |  |  |  | 0.27 |
| Ferritin (μg/liter), median (IQR) |  |  |  |  |  |  |  |  |  |  |  |  |
| Day 1 | 285.0 (241.6–343.4) | |  | 263.3 (228.5–304.8) | |  | 23.7 (-4.2 to 52.5) | |  |  |  | 0.09 |
| Day 3 | 304.9 (256.7–361.8) | |  | 264.5 (222.8–320.7) | |  | 33.4 (1.1 to 64.7) | |  |  |  | 0.04 |
| Day 5 | 256.1 (219.5–289.0) | |  | 262.7 (221.9–368.5) | |  | -17.5 (-52.9 to 12.1) | |  |  |  | 0.23 |
| Day 7 | 233.6 (218.4–277.2) | |  | 306.4 (254.9–381.5) | |  | -73.9 (-117.5 to -34.7) | |  |  |  | <0.001 |
| LDH (U/liter), median (IQR) |  |  |  |  |  |  |  |  |  |  |  |  |
| Day 1 | 423.0 (372.3–482.0) | |  | 446.0 (409.5–515.8) | |  | -29.0 (-65.0 to 6.0) | |  |  |  | 0.11 |
| Day 3 | 669.0 (561.5–801.3) | |  | 722.0 (636.8–840.3) | |  | -59.0 (-120.0 to 5.0) | |  |  |  | 0.08 |
| Day 5 | 724.0 (617.0–807.0) | |  | 772.0 (676.5–898.8) | |  | -54.5 (-120.0 to 9.0) | |  |  |  | 0.08 |
| LDH (day 7), mean (±SD) | 660.3 ± 134.8 | |  | 820.2 ± 136.1 | |  | -159.9 (-231.6 to -88.3) | |  |  |  | <0.001 |
| AST |  |  |  |  |  |  |  |  |  |  |  |  |
| Day 1 |  | |  |  | |  |  |  |  |  |  |  |
| ≤39 U/liter, n (%) | 43 (97.7) | |  | 44 (95.7) | |  | – | |  | – |  | 0.58 |
| >39 U/liter, n (%) | 1 (2.3) | |  | 2 (4.3) | |  |  |  |  |  |  |  |
| Day 3 |  | |  |  | |  |  |  |  |  |  |  |
| ≤39 U/liter, n (%) | 29 (65.9) | |  | 25 (54.3) | |  | ARR 11.56 (-8.52 to 31.65) | |  | NNT=9 (Helpful >3.2) (Harmful >11.7) |  | 0.26 |
| >39 U/liter, n (%) | 15 (34.1) | |  | 21 (45.7) | |  |  |  |  |  |  |  |
| Day 5 |  | |  |  | |  |  |  |  |  |  |  |
| ≤39 U/liter, n (%) | 24 (58.5) | |  | 18 (39.1) | |  | ARR 19.41 (-1.24 to 40.05) | |  | NNT=6 (Helpful >2.5) (Harmful >80.6) |  | 0.07 |
| >39 U/liter, n (%) | 17 (41.5) | |  | 28 (60.9) | |  |  |  |  |  |  |  |
| Day 7 |  | |  |  | |  |  |  |  |  |  |  |
| ≤39 U/liter, n (%) | 17 (73.9) | |  | 12 (31.6) | |  | ARR 42.33 (19.09 to 65.58) | |  | NNT=3 (1.5 to 5.2) |  | 0.001 |
| >39 U/liter, n (%) | 6 (26.1) | |  | 26 (68.4) | |  |  |  |  |  |  |  |
| ALT |  |  |  |  |  |  |  |  |  |  |  |  |
| Day 1 |  | |  |  | |  |  |  |  |  |  |  |
| ≤36 U/liter, n (%) | 43 (97.7) | |  | 44 (95.7) | |  | – | |  | – |  | 0.58 |
| >36 U/liter, n (%) | 1 (2.3) | |  | 2 (4.3) | |  |  |  |  |  |  |  |
| Day 3 |  | |  |  | |  |  |  |  |  |  |  |
| ≤36 U/liter, n (%) | 25 (56.8) | |  | 33 (71.7) | |  | ARI 14.92 (-4.66 to 34.50) | |  | NNH=7 (Harmful >2.9) (Helpful >21.4) |  | 0.14 |
| >36 U/liter, n (%) | 19 (43.2) | |  | 13 (28.3) | |  |  |  |  |  |  |  |
| Day 5 |  | |  |  | |  |  |  |  |  |  |  |
| ≤36 U/liter, n (%) | 24 (58.5) | |  | 19 (41.3) | |  | ARR 17.23 (-3.50 to 37.97) | |  | NNT=6 (Helpful >2.6) (Harmful >28.6) |  | 0.11 |
| >36 U/liter, n (%) | 17 (41.5) | |  | 27 (58.7) | |  |  |  |  |  |  |  |
| Day 7 |  | |  |  | |  |  |  |  |  |  |  |
| ≤36 U/liter, n (%) | 15 (65.2) | |  | 14 (36.8) | |  | ARR 28.38 (3.59 to 53.16) | |  | NNT=4 (1.9 to 27.8) |  | 0.03 |
| >36 U/liter, n (%) | 8 (34.8) | |  | 24 (63.2) | |  |  |  |  |  |  |  |
| CRP |  |  |  |  |  |  |  |  |  |  |  |  |
| Day 1 |  | |  |  | |  |  |  |  |  |  |  |
| ≤35 mg/liter, n (%) | 29 (65.9) | |  | 36 (78.3) | |  | – | |  | – |  | 0.19 |
| >35 mg/liter, n (%) | 15 (34.1) | |  | 10 (21.7) | |  |  |  |  |  |  |  |
| Day 3 |  | |  |  | |  |  |  |  |  |  |  |
| ≤35 mg/liter, n (%) | 38 (86.4) | |  | 34 (73.9) | |  | ARR 12.45 (-3.79 to 28.69) | |  | NNT=9 (Helpful >3.5) (Harmful >26.4) |  | 0.14 |
| >35 mg/liter, n (%) | 6 (13.6) | |  | 12 (26.1) | |  |  |  |  |  |  |  |
| Day 5 |  | |  |  | |  |  |  |  |  |  |  |
| ≤35 mg/liter, n (%) | 41 (100.0) | |  | 36 (78.3) | |  | ARR 21.74 (9.82 to 33.66) | |  | NNT=5 (3.0 to 10.2) |  | 0.002 |
| >35 mg/liter, n (%) | 0 (0.0) | |  | 10 (21.7) | |  |  |  |  |  |  |  |
| Day 7 |  | |  |  | |  |  |  |  |  |  |  |
| ≤35 mg/liter, n (%) | 23 (100.0) | |  | 24 (63.2) | |  | ARR 36.84 (21.51 to 52.18) | |  | NNT=3 (1.9 to 4.7) |  | <0.001 |
| >35 mg/liter, n (%) | 0 (0.0) | |  | 14 (36.8) | |  |  |  |  |  |  |  |
| ESR |  |  |  |  |  |  |  |  |  |  |  |  |
| Day 1 |  | |  |  | |  |  |  |  |  |  |  |
| ≤41 mm/hr, n (%) | 31 (70.5) | |  | 36 (78.3) | |  | – | |  | – |  | 0.40 |
| >41 mm/hr, n (%) | 13 (29.5) | |  | 10 (21.7) | |  |  |  |  |  |  |  |
| Day 3 |  | |  |  | |  |  |  |  |  |  |  |
| ≤41 mm/hr, n (%) | 37 (84.1) | |  | 29 (63.0) | |  | ARR 21.05 (3.40 to 38.69) | |  | NNT=5 (2.6 to 29.4) |  | 0.02 |
| >41 mm/hr, n (%) | 7 (15.9) | |  | 17 (37.0) | |  |  |  |  |  |  |  |
| Day 5 |  | |  |  | |  |  |  |  |  |  |  |
| ≤41 mm/hr, n (%) | 41 (100.0) | |  | 34 (73.9) | |  | ARR 26.09 (13.40 to 38.78) | |  | NNT=4 (2.6 to 7.5) |  | <0.001 |
| >41 mm/hr, n (%) | 0 (0.0) | |  | 12 (26.1) | |  |  |  |  |  |  |  |
| Day 7 |  | |  |  | |  |  |  |  |  |  |  |
| ≤41 mm/hr, n (%) | 23 (100.0) | |  | 26 (68.4) | |  | ARR 31.58 (16.80 to 46.36) | |  | NNT=4 (2.2 to 6.0) |  | 0.003 |
| >41 mm/hr, n (%) | 0 (0.0) | |  | 12 (31.6) | |  |  |  |  |  |  |  |
|  |  |  |  |  |  |  |  |  |  |  |  |  |
|  | **ICU** | | | | |  | **Difference (95% CI)** | |  | **NNT or NNH (95% CI)** |  | **P Value** |
|  | **Spirulina (n=47)** | |  | **Control (n=52)** | |  |  | |  |  |  |  |
| Creatinine (mg/dl), median (IQR) |  |  |  |  |  |  |  |  |  |  |  |  |
| Day 1 | 1.2 (1.0–1.4) | |  | 1.3 (1.1–1.4) | |  | -0.08 (-0.16 to 0.01) | |  |  |  | 0.08 |
| Day 3 | 1.2 (1.1–1.3) | |  | 1.3 (1.2–1.4) | |  | -0.11 (-0.15 to -0.06) | |  |  |  | <0.001 |
| Day 5 | 1.2 (1.1–1.3) | |  | 1.3 (1.2–1.4) | |  | -0.15 (-0.23 to -0.07) | |  |  |  | <0.001 |
| Day 7 | 1.1 (1.1–1.3) | |  | 1.3 (1.2–1.4) | |  | -0.15 (-0.25 to -0.07) | |  |  |  | <0.001 |
| D-dimer(ng/ml), median (IQR) |  |  |  |  |  |  |  |  |  |  |  |  |
| D-dimer (day 1), mean (±SD) | 763.1 ± 295.0 | |  | 762.0 ± 219.1 | |  | 1.2 (-103.6 to 105.9) | |  |  |  | 0.98 |
| Day 3 | 781.0 (640.0–888.0) | |  | 809.5 (667.0–939.5) | |  | -39.0 (-117.0 to 43.0) | |  |  |  | 0.30 |
| Day 5 | 671.0 (614.0–805.0) | |  | 749.0 (656.0–963.5) | |  | -83.0 (-160.0 to -14.0) | |  |  |  | 0.02 |
| Day 7 | 686.5 (582.0–782.5) | |  | 756.0 (633.5–891.0) | |  | -85.0 (-166.0 to -10.0) | |  |  |  | 0.03 |
| Ferritin (μg/liter), median (IQR) |  |  |  |  |  |  |  |  |  |  |  |  |
| Day 1 | 487.3 (379.2–631.4) | |  | 540.1 (456.2–778.3) | |  | -72.2 (-157.9 to 3.9) | |  |  |  | 0.06 |
| Day 3 | 484.3 (418.0–595.5) | |  | 527.0 (445.0–683.8) | |  | -49.3 (-118.2 to 10.2) | |  |  |  | 0.11 |
| Day 5 | 436.9 (342.6–502.9) | |  | 466.0 (387.8–620.6) | |  | -57.2 (-117.4 to 0) | |  |  |  | 0.05 |
| Day 7 | 409.7 (309.1–458.0) | |  | 434.1 (382.9–576.0) | |  | -66.5 (-131.5 to -7.9) | |  |  |  | 0.02 |
| LDH (U/liter), median (IQR) |  |  |  |  |  |  |  |  |  |  |  |  |
| Day 1 | 892.0 (726.0–974.0) | |  | 866.0 (727.0–1017.3) | |  | 7.5 (-70.0 to 84.0) | |  |  |  | 0.81 |
| Day 3 | 847.0 (744.0–1000.0) | |  | 796.0 (697.5–923.0) | |  | 53.5 (-17.0 to 121.0) | |  |  |  | 0.12 |
| Day 5 | 842.0 (768.0–919.3) | |  | 785.0 (660.0–882.0) | |  | 57.0 (-12.0 to 125.0) | |  |  |  | 0.10 |
| Day 7 | 781.0 (676.5–950.3) | |  | 716.0 (639.5–828.0) | |  | 62.0 (-9.0 to 137.0) | |  |  |  | 0.08 |
| AST |  |  |  |  |  |  |  |  |  |  |  |  |
| Day 1 |  | |  |  | |  |  |  |  |  |  |  |
| ≤39 U/liter, n (%) | 5 (10.6) | |  | 4 (7.7) | |  | – | |  | – |  | 0.73 |
| >39 U/liter, n (%) | 42 (89.4) | |  | 48 (92.3) | |  |  |  |  |  |  |  |
| Day 3 |  | |  |  | |  |  |  |  |  |  |  |
| ≤39 U/liter, n (%) | 1 (2.1) | |  | 5 (10.4) | |  | ARI 8.29 (-1.29 to 17.87) | |  | NNH=13 (Harmful >5.6) (Helpful >77.7) |  | 0.10 |
| >39 U/liter, n (%) | 46 (97.9) | |  | 43 (89.6) | |  |  |  |  |  |  |  |
| Day 5 |  | |  |  | |  |  |  |  |  |  |  |
| ≤39 U/liter, n (%) | 2 (5.0) | |  | 3 (6.7) | |  | ARI 1.67 (-8.27 to 11.60) | |  | NNH=60 (Harmful >8.6) (Helpful >12.1) |  | 0.74 |
| >39 U/liter, n (%) | 38 (95.0) | |  | 42 (93.3) | |  |  |  |  |  |  |  |
| Day 7 |  | |  |  | |  |  |  |  |  |  |  |
| ≤39 U/liter, n (%) | 6 (15.8) | |  | 5 (12.2) | |  | ARR 3.59 (-11.73 to 18.92) | |  | NNT=28 (Helpful >5.3) (Harmful >8.5) |  | 0.64 |
| >39 U/liter, n (%) | 32 (84.2) | |  | 36 (87.8) | |  |  |  |  |  |  |  |
| ALT |  |  |  |  |  |  |  |  |  |  |  |  |
| Day 1 |  | |  |  | |  |  |  |  |  |  |  |
| ≤36 U/liter, n (%) | 3 (6.4) | |  | 7 (13.5) | |  | – | |  | – |  | 0.32 |
| >36 U/liter, n (%) | 44 (93.6) | |  | 45 (86.5) | |  |  |  |  |  |  |  |
| Day 3 |  | |  |  | |  |  |  |  |  |  |  |
| ≤36 U/liter, n (%) | 5 (10.6) | |  | 3 (6.2) | |  | ARR 4.39 (-6.77 to 15.55) | |  | NNT=23 (Helpful >6.4) (Harmful >14.8) |  | 0.44 |
| >36 U/liter, n (%) | 42 (89.4) | |  | 45 (93.8) | |  |  |  |  |  |  |  |
| Day 5 |  | |  |  | |  |  |  |  |  |  |  |
| ≤36 U/liter, n (%) | 2 (5.0) | |  | 3 (6.7) | |  | ARI 1.67 (-8.27 to 11.60) | |  | NNH=60 (Harmful >8.6) (Helpful >12.1) |  | 0.74 |
| >36 U/liter, n (%) | 38 (95.0) | |  | 42 (93.3) | |  |  |  |  |  |  |  |
| Day 7 |  | |  |  | |  |  |  |  |  |  |  |
| ≤36 U/liter, n (%) | 3 (7.9) | |  | 5 (12.2) | |  | ARI 4.30 (-8.88 to 17.49) | |  | NNH=24 (Harmful >5.7) (Helpful >11.3) |  | 0.53 |
| >36 U/liter, n (%) | 35 (92.1) | |  | 36 (87.8) | |  |  |  |  |  |  |  |
| CRP |  |  |  |  |  |  |  |  |  |  |  |  |
| Day 1 |  | |  |  | |  |  |  |  |  |  |  |
| ≤35 mg/liter, n (%) | 12 (25.5) | |  | 22 (42.3) | |  | – | |  | – |  | 0.08 |
| >35 mg/liter, n (%) | 35 (74.5) | |  | 30 (57.7) | |  |  |  |  |  |  |  |
| Day 3 |  | |  |  | |  |  |  |  |  |  |  |
| ≤35 mg/liter, n (%) | 19 (40.4) | |  | 18 (37.5) | |  | ARR 2.93 (-16.68 to 22.53) | |  | NNT=35 (Helpful >4.4) (Harmful >6.0) |  | 0.77 |
| >35 mg/liter, n (%) | 28 (59.6) | |  | 30 (62.5) | |  |  |  |  |  |  |  |
| Day 5 |  | |  |  | |  |  |  |  |  |  |  |
| ≤35 mg/liter, n (%) | 26 (65.0) | |  | 24 (53.3) | |  | ARR 11.67 (-9.09 to 32.43) | |  | NNT=9 (Helpful >3.1) (Harmful >11.0) |  | 0.38 |
| >35 mg/liter, n (%) | 14 (35.0) | |  | 21 (46.7) | |  |  |  |  |  |  |  |
| Day 7 |  | |  |  | |  |  |  |  |  |  |  |
| ≤35 mg/liter, n (%) | 20 (52.6) | |  | 25 (61.0) | |  | ARI 8.34 (-13.45 to 30.14) | |  | NNH=12 (Harmful >3.3) (Helpful >7.4) |  | 0.45 |
| >35 mg/liter, n (%) | 18 (47.4) | |  | 16 (39.0) | |  |  |  |  |  |  |  |
| ESR |  |  |  |  |  |  |  |  |  |  |  |  |
| Day 1 |  | |  |  | |  |  |  |  |  |  |  |
| ≤41 mm/hr, n (%) | 12 (25.5) | |  | 18 (34.6) | |  | – | |  | – |  | 0.33 |
| >41 mm/hr, n (%) | 35 (74.5) | |  | 34 (65.4) | |  |  |  |  |  |  |  |
| Day 3 |  | |  |  | |  |  |  |  |  |  |  |
| ≤41 mm/hr, n (%) | 20 (42.6) | |  | 16 (33.3) | |  | ARR 9.22 (-10.21 to 28.65) | |  | NNT=11 (Helpful >3.5) (Harmful >9.8) |  | 0.35 |
| >41 mm/hr, n (%) | 27 (57.4) | |  | 32 (66.7) | |  |  |  |  |  |  |  |
| Day 5 |  | |  |  | |  |  |  |  |  |  |  |
| ≤41 mm/hr, n (%) | 25 (62.5) | |  | 19 (42.2) | |  | ARR 20.28 (-0.54 to 41.09) | |  | NNT=5 (Helpful >2.4) (Harmful >185.5) |  | 0.06 |
| >41 mm/hr, n (%) | 15 (37.5) | |  | 26 (57.8) | |  |  |  |  |  |  |  |
| Day 7 |  | |  |  | |  |  |  |  |  |  |  |
| ≤41 mm/hr, n (%) | 31 (81.6) | |  | 21 (51.2) | |  | ARR 30.36 (10.71 to 50.01) | |  | NNT=4 (2.0 to 9.3) |  | 0.008 |
| >41 mm/hr, n (%) | 7 (18.4) | |  | 20 (48.8) | |  |  |  |  |  |  |  |
| Note: Plus-minus values are means ± standard deviation (SD). IQR denotes the interquartile range [median (25^th^ percentile–75^th^ percentile)]. The number of patients (for all variables): in the non-ICU subgroup intervention group (first day n=44, third day n=44, fifth day n=41, and seventh day n=23); control group (first day n=46, third day n=46, fifth day n=46, and seventh day n=38), and the ICU subgroup: intervention group (first day n=47, third day n=47, fifth day n=40, and seventh day n=38); control group (first day n=52, third day n=48, fifth day n=45, and seventh day n=41). Differences were expressed as the median difference (Hodges–Lehmann estimate), mean difference or number needed to treat (NNT) with absolute risk reduction (ARR), the number needed to harm (NNH) with absolute risk increase (ARI), and 95% confidence intervals. To calculate NNT, ARR, NNH, and ARI for AST, ALT, CRP, and ESR variables, the events were defined as follows: AST (Good Outcome: ≤39 U/liter and Bad Outcome: >39 U/liter), ALT (Good Outcome: ≤36 U/liter and Bad Outcome: >36 U/liter), CRP (Good Outcome: ≤35 mg/liter and Bad Outcome: >35 mg/liter), and ESR (Good Outcome: ≤41 mm/hr and Bad Outcome: >41 mm/hr).  Abbreviations: LDH, lactate dehydrogenase; AST, aspartate aminotransferase; ALT, alanine aminotransferase; CRP, C-reactive protein; ESR, erythrocyte sedimentation rate. | | | | | | | | | | | | |
