# Supplementary material for: Effect of high-dose Spirulina supplementation on hospitalized adults with COVID-19: a randomized controlled trial
Source: Front Immunol. 2024 Apr 8;15:1332425. doi: 10.3389/fimmu.2024.1332425 (PMC11036872; doi:10.3389/fimmu.2024.1332425)
Supplement: Supplementary file 2 [file Table_5.docx]

| TABLE 5 Monitoring of immune mediators before and after treatment. | | | | | | | | | | | |
| --- | --- | --- | --- | --- | --- | --- | --- | --- | --- | --- | --- |
|  |  | | | | |  | **Difference (95% CI)** | |  |  | **P Value** |
|  | **Before treatment** | |  | **After treatment** | |  |  | |  |  |  |
| Immune mediators |  |  |  |  |  |  |  |  |  |  |  |
| Pro-inflammatory cytokines |  |  |  |  |  |  |  |  |  |  |  |
| IL-6 (ng/ml) |  |  |  |  |  |  |  |  |  |  |  |
| Non-ICU spirulina, median (IQR) | 90.2 (70.7–146.3) | |  | 65.3 (47.4–79.7) | |  | -28.8 (-57.7 to -17.7) | |  |  | <0.001 |
| Non-ICU control, median (IQR) | 79.4 (63.6–118.9) | |  | 91.0 (76.7–124.0) | |  | 10.3 (-8.1 to 24.2) | |  |  | 0.24 |
| ICU spirulina, median (IQR) | 227.6 (119.6–318.6) | |  | 126.7 (90.4–182.7) | |  | -77.1 (-124.8 to -32.4) | |  |  | <0.001 |
| ICU control, median (IQR) | 243.0 (150.0–336.7) | |  | 188.4 (135.1–231.8) | |  | -46.2 (-91.8 to 18.3) | |  |  | 0.01 |
| TNF-α (ng/ml) |  |  |  |  |  |  |  |  |  |  |  |
| Non-ICU spirulina, median (IQR) | 51.8 (38.8–68.8) | |  | 34.4 (28.5–42.5) | |  | -14.2 (-17.7 to -8.9) | |  |  | <0.001 |
| Non-ICU control, median (IQR) | 37.3 (27.6–63.2) | |  | 47.2 (36.6–59.6) | |  | 1.9 (-2.7 to 10.5) | |  |  | 0.31 |
| ICU spirulina, mean (±SD) | 93.1 ± 34.4 | |  | 74.3 ± 22.2 | |  | -18.8 (-28.0 to -9.7) | |  |  | <0.001 |
| ICU control, mean (±SD) | 78.6 ± 22.6 | |  | 71.6 ± 17.6 | |  | -7.0 (-15.1 to 1.05) | |  |  | 0.09 |
| Anti-inflammatory cytokine |  |  |  |  |  |  |  |  |  |  |  |
| IL-10 (ng/ml) |  |  |  |  |  |  |  |  |  |  |  |
| Non-ICU spirulina, median (IQR) | 8.1 (7.1–9.7) | |  | 7.6 (7.2–8.3) | |  | -0.6 (-0.9 to -0.2) | |  |  | 0.010 |
| Non-ICU control, median (IQR) | 7.7 (6.6–8.9) | |  | 8.9 (7.6–9.7) | |  | 0.5 (0.1 to 1.5) | |  |  | 0.03 |
| ICU spirulina, mean (±SD) | 13.7 ± 4.9 | |  | 12.3 ± 2.9 | |  | -1.5 (-2.6 to -0.4) | |  |  | 0.01 |
| ICU control, mean (±SD) | 14.0 ± 3.2 | |  | 12.4 ± 2.4 | |  | -1.6 (-2.6 to -0.7) | |  |  | 0.001 |
| Inflammatory cytokine |  | |  |  | |  |  | |  |  |  |
| IFN-γ (ng/ml) |  |  |  |  |  |  |  |  |  |  |  |
| Non-ICU spirulina, median (IQR) | 43.1 (37.0–51.7) | |  | 71.8 (58.8–93.9) | |  | 28.5 (22.0 to 39.1) | |  |  | <0.001 |
| Non-ICU control, mean (±SD) | 45.6 ± 11.5 | |  | 56.8 ± 13.9 | |  | 11.2 (7.4 to 14.9) | |  |  | <0.001 |
| ICU spirulina, mean (±SD) | 55.5 ± 21.9 | |  | 97.3 ± 34.4 | |  | 41.9 (32.7 to 51.0) | |  |  | <0.001 |
| ICU control, median (IQR) | 55.3 (43.0–63.8) | |  | 62.2 (50.8–67.0) | |  | 6.2 (-0.5 to 11.3) | |  |  | 0.12 |
| Chemokines |  |  |  |  |  |  |  |  |  |  |  |
| IP-10 (ng/ml) |  |  |  |  |  |  |  |  |  |  |  |
| Non-ICU spirulina, median (IQR) | 187.5 (150.7–250.9) | |  | 178.5 (160.0–198.3) | |  | -22.0 (-31.4 to 8.6) | |  |  | 0.02 |
| Non-ICU control, median (IQR) | 193.7 (168.3–211.6) | |  | 268.8 (229.5–367.4) | |  | 93.7 (62.7 to 118.0) | |  |  | <0.001 |
| ICU spirulina, mean (±SD) | 513.1 ± 197.2 | |  | 495.1 ± 115.3 | |  | -18.0 (-62.6 to 26.7) | |  |  | 0.42 |
| ICU control, mean (±SD) | 544.9 ± 153.7 | |  | 475.4 ± 108.3 | |  | -69.5 (-114.7 to -24.3) | |  |  | 0.004 |
| MIP-1α (ng/ml) |  |  |  |  |  |  |  |  |  |  |  |
| Non-ICU spirulina, mean (±SD) | 23.5 ± 10.2 | |  | 22.6 ± 7.3 | |  | -0.9 (-4.7 to 3.0) | |  |  | 0.66 |
| Non-ICU control, median (IQR) | 20.3 (16.3–23.9) | |  | 24.3 (20.9–26.8) | |  | 3.6 (0.6 to 6.3) | |  |  | 0.04 |
| ICU spirulina, mean (±SD) | 20.4 ± 7.8 | |  | 18.0 ± 4.5 | |  | -2.4 (-4.1 to -0.6) | |  |  | 0.009 |
| ICU control, mean (±SD) | 20.8 ± 5.2 | |  | 19.3 ± 4.0 | |  | -1.5 (-3.0 to 0.05) | |  |  | 0.06 |
| MCP-1 (ng/ml) |  |  |  |  |  |  |  |  |  |  |  |
| Non-ICU spirulina, median (IQR) | 195.6 (178.8–223.2) | |  | 195.3 (187.4– 205.2) | |  | -2.2 (-9.0 to 2.9) | |  |  | 0.36 |
| Non-ICU control, median (IQR) | 195.1 (165.0–216.7) | |  | 206.1 (183.0–224.1) | |  | 15.9 (3.9 to 28.2) | |  |  | 0.21 |
| ICU spirulina, median (IQR) | 286.6 (229.5–361.8) | |  | 266.5 (238.7–296.3) | |  | -28.1 (-50.3 to -0.3) | |  |  | 0.006 |
| ICU control, mean (±SD) | 303.9 ± 55.3 | |  | 276.0 ± 41.9 | |  | -27.9 (-44.3 to -11.5) | |  |  | 0.001 |
|  |  |  |  |  |  |  |  |  |  |  |  |
| Note: Plus-minus values are means ± standard deviation (SD). IQR denotes the interquartile range [median (25^th^ percentile–75^th^ percentile)]. The number of patients (for all variables): in the non-ICU subgroup: intervention group (n=44); control group (n=42), and the ICU subgroup: intervention group (n=47); control group (n=37). Differences were expressed as the median difference or mean difference and 95% confidence intervals.  Abbreviations: IL-6, interleukin-6; TNF-α, tumor necrosis factor alpha; IL-10, interleukin-10; IFN-γ, interferon-γ; IP-10, interferon gamma-induced protein 10; MIP-1α, macrophage inflammatory protein 1α; MCP-1, monocyte chemotactic protein 1. | | | | | | | | | | | |
