# Supplementary material for: Effect of high-dose Spirulina supplementation on hospitalized adults with COVID-19: a randomized controlled trial
Source: Front Immunol. 2024 Apr 8;15:1332425. doi: 10.3389/fimmu.2024.1332425 (PMC11036872; doi:10.3389/fimmu.2024.1332425)
Supplement: Supplementary file 3 [file Table_6.docx]

| TABLE 6 Monitoring of immune mediators in deceased and surviving patients. | | | | | | | | | | | |
| --- | --- | --- | --- | --- | --- | --- | --- | --- | --- | --- | --- |
|  | **Non-ICU** | | | | |  | **Difference (95% CI)** | |  |  | **P Value** |
|  | **Death (n=4)** | |  | **Survival (n=42)** | |  |  | |  |  |  |
| Immune mediators |  |  |  |  |  |  |  |  |  |  |  |
| Pro-inflammatory cytokines |  |  |  |  |  |  |  |  |  |  |  |
| IL-6 (ng/ml), median (IQR) |  |  |  |  |  |  |  |  |  |  |  |
| Day 1 | 55.2 (37.0–82.3) | |  | 79.4 (63.6–118.9) | |  | 26.2 (-6.1 to 80.0) | |  |  | 0.09 |
| Day 3 | 235.6 (123.0–354.7) | |  | 87.8 (53.8–115.3) | |  | -141.0 (-273.7 to -23.6) | |  |  | 0.02 |
| Day 5 | 247.3 (222.1–410.3) | |  | 112.5 (84.4–131.1) | |  | -143.2 (-321.2 to -94.6) | |  |  | 0.005 |
| Day 7 | ̶ | |  | 95.9 (79.4–126.8) | |  | ̶ | |  |  | ̶ |
| TNF-α (ng/ml), median (IQR) |  |  |  |  |  |  |  |  |  |  |  |
| Day 1 | 72.0 (59.1–135.3) | |  | 37.3 (27.6–63.2) | |  | -36.5 (-90.6 to -7.1) | |  |  | 0.02 |
| Day 3 | 114.8 (85.4–167.5) | |  | 49.1 (34.9–61.4) | |  | -63.4 (-114.9 to -34.8) | |  |  | <0.001 |
| Day 5 | 175.7 (167.3–221.9) | |  | 44.5 (34.0–58.9) | |  | -133.7 (-173.8 to -115.9) | |  |  | <0.001 |
| Day 7 | ̶ | |  | 47.8 (37.7–60.8) | |  | ̶ | |  |  | ̶ |
| Anti-inflammatory cytokine |  |  |  |  |  |  |  |  |  |  |  |
| IL-10 (ng/ml), median (IQR) |  |  |  |  |  |  |  |  |  |  |  |
| Day 1 | 8.1 (7.7–10.1) | |  | 7.7 (6.6–8.9) | |  | -0.6 (-2.6 to 0.9) | |  |  | 0.34 |
| Day 3 | 17.9 (11.2–19.9) | |  | 8.4 (7.1–9.2) | |  | -9.3 (-11.8 to -2.2) | |  |  | <0.001 |
| Day 5 | 20.3 (19.6–26.5) | |  | 8.6 (7.7–9.8) | |  | -11.9 (-18.3 to -10.3) | |  |  | 0.001 |
| Day 7 | ̶ | |  | 9.1 (7.8–9.8) | |  | ̶ | |  |  | ̶ |
| Inflammatory cytokine |  | |  |  | |  |  | |  |  |  |
| IFN-γ (ng/ml), median (IQR) |  |  |  |  |  |  |  |  |  |  |  |
| Day 1 | 53.1 (37.8–67.0) | |  | 45.5 (36.0–54.1) | |  | -8.0 (-22.7 to 9.7) | |  |  | 0.46 |
| Day 3 | 92.2 (65.3–105.3) | |  | 56.7 (46.7–66.7) | |  | -33.6 (-50.3 to -8.0) | |  |  | 0.01 |
| Day 5 | 147.2 (136.3–155.5) | |  | 50.6 (40.6–65.6) | |  | -95.1 (-108.1 to -79.3) | |  |  | <0.001 |
| Day 7 | ̶ | |  | 55.0 (45.9–66.3) | |  | ̶ | |  |  | ̶ |
| Chemokines |  |  |  |  |  |  |  |  |  |  |  |
| IP-10 (ng/ml), median (IQR) |  |  |  |  |  |  |  |  |  |  |  |
| Day 1 | 173.9 (157.3–208.2) | |  | 193.7 (168.3–211.6) | |  | 12.9 (-26.6 to 47.0) | |  |  | 0.56 |
| Day 3 | 449.3 (274.0–454.8) | |  | 232.1 (198.2–259.3) | |  | -203.4 (-250.6 to -14.7) | |  |  | 0.02 |
| Day 5 | 575.2 (486.2–733.0) | |  | 250.6 (209.2–284.4) | |  | -315.3 (-477.7 to -222.3) | |  |  | <0.001 |
| Day 7 | ̶ | |  | 290.7 (240.9–389.8) | |  | ̶ | |  |  | ̶ |
| MIP-1α (ng/ml), median (IQR) |  |  |  |  |  |  |  |  |  |  |  |
| Day 1 | 19.8 (17.6–22.0) | |  | 20.3 (16.3–23.9) | |  | 0.2 (-4.3 to 5.1) | |  |  | 0.93 |
| Day 3 | 31.7 (17.1–49.5) | |  | 23.1 (20.7–26.5) | |  | -7.1 (-26.6 to 6.6) | |  |  | 0.26 |
| Day 5 | 32.6 (28.6–42.2) | |  | 24.6 (22.5– 27.9) | |  | -7.8 (-17.5 to -1.8) | |  |  | 0.02 |
| Day 7 | ̶ | |  | 24.7 (20.9–26.8) | |  | ̶ | |  |  | ̶ |
| MCP-1 (ng/ml), median (IQR) |  |  |  |  |  |  |  |  |  |  |  |
| Day 1 | 184.2 (179.2–208.4) | |  | 195.1 (165.0–216.7) | |  | 7.3 (-27.0 to 42.7) | |  |  | 0.73 |
| Day 3 | 326.0 (222.6–376.2) | |  | 192.6 (176.0–221.1) | |  | -124.0 (-177.2 to -15.0) | |  |  | 0.02 |
| Day 5 | 351.9 (322.5–458.1) | |  | 207.8 (193.3–231.8) | |  | -141.6 (-254.6 to -102.8) | |  |  | 0.004 |
| Day 7 | ̶ | |  | 207.6 (183.4–228.9) | |  | ̶ | |  |  | ̶ |
|  |  |  |  |  |  |  |  |  |  |  |  |
|  | **ICU** | | | | |  | **Difference (95% CI)** | |  |  | **P Value** |
|  | **Death (n=15)** | |  | **Survival (n=37)** | |  |  | |  |  |  |
| Immune mediators |  |  |  |  |  |  |  |  |  |  |  |
| Pro-inflammatory cytokines |  |  |  |  |  |  |  |  |  |  |  |
| IL-6 (ng/ml), median (IQR) |  |  |  |  |  |  |  |  |  |  |  |
| Day 1 | 164.5 (110.0–287.4) | |  | 243.0 (150.0–336.7) | |  | 57.0 (-16.4 to 128.7) | |  |  | 0.18 |
| Day 3 | 284.7 (227.9–367.6) | |  | 214.6 (158.3–275.4) | |  | -71.8 (-145.3 to 9.5) | |  |  | 0.07 |
| Day 5 | 334.8 (217.3–436.5) | |  | 194.9 (136.5–284.9) | |  | -118.8 (-230.4 to -10.6) | |  |  | 0.03 |
| Day 7 | 230.4 (171.5–273.0) | |  | 188.4 (135.1–231.8) | |  | -37.8 (-106.1 to 37.8) | |  |  | 0.31 |
| TNF-α (ng/ml), median (IQR) |  |  |  |  |  |  |  |  |  |  |  |
| Day 1 | 95.1 (61.9–128.5) | |  | 73.1 (62.5–92.6) | |  | -20.2 (-44.2 to 2.0) | |  |  | 0.09 |
| Day 3 | 117.5 (87.1–136.5) | |  | 83.3 (62.4–95.3) | |  | -32.6 (-54.3 to -12.3) | |  |  | 0.003 |
| Day 5 | 137.4 (117.9–158.1) | |  | 72.8 (55.7–90.1) | |  | -65.0 (-84.8 to -43.0) | |  |  | <0.001 |
| Day 7 | 155.6 (107.4–166.1) | |  | 71.3 (57.2–86.2) | |  | -80.9 (-100.6 to -36.6) | |  |  | <0.001 |
| Anti-inflammatory cytokine |  |  |  |  |  |  |  |  |  |  |  |
| IL-10 (ng/ml), median (IQR) |  |  |  |  |  |  |  |  |  |  |  |
| Day 1 | 13.3 (11.5–17.1) | |  | 14.1 (11.5–16.2) | |  | -0.1 (-2.3 to 1.8) | |  |  | 0.85 |
| Day 3 | 16.7 (15.2–21.0) | |  | 14.3 (10.8–15.4) | |  | -3.8 (-6.7 to -1.4) | |  |  | 0.001 |
| Day 5 | 22.7 (16.5–27.1) | |  | 12.4 (11.0–14.9) | |  | -9.1 (-13.0 to -4.4) | |  |  | <0.001 |
| Day 7 | 20.4 (15.2–21.0) | |  | 12.8 (10.3–13.6) | |  | -7.4 (-9.6 to -2.5) | |  |  | 0.004 |
| Inflammatory cytokine |  | |  |  | |  |  | |  |  |  |
| IFN-γ (ng/ml), median (IQR) |  |  |  |  |  |  |  |  |  |  |  |
| Day 1 | 82.3 (56.9–135.9) | |  | 55.3 (43.0–63.8) | |  | -31.0 (-62.6 to -11.7) | |  |  | <0.001 |
| Day 3 | 114.2 (43.8–188.5) | |  | 63.4 (47.5–74.0) | |  | -44.2 (-90.3 to 4.4) | |  |  | 0.10 |
| Day 5 | 133.8 (94.9–199.3) | |  | 58.5 (51.3–72.3) | |  | -74.0 (-124.5 to -40.5) | |  |  | <0.001 |
| Day 7 | 166.3 (121.4–190.9) | |  | 62.2 (50.8–67.0) | |  | -102.4 (-132.1 to -60.2) | |  |  | <0.001 |
| Chemokines |  |  |  |  |  |  |  |  |  |  |  |
| IP-10 (ng/ml), median (IQR) |  |  |  |  |  |  |  |  |  |  |  |
| Day 1 | 501.5 (402.5–716.0) | |  | 564.4 (414.9–670.3) | |  | -11.9 (-130.9 to 103.7) | |  |  | 0.83 |
| Day 3 | 649.7 (549.8–800.3) | |  | 532.9 (408.3–598.9) | |  | -140.5 (-263.7 to -53.9) | |  |  | 0.008 |
| Day 5 | 815.6 (594.2–1053.3) | |  | 484.8 (410.4–585.2) | |  | -302.2 (-521.0 to -107.3) | |  |  | 0.003 |
| Day 7 | 755.2 (563.5–923.3) | |  | 472.2 (392.6–523.7) | |  | -279.5 (-457.5 to -84.0) | |  |  | 0.009 |
| MIP-1α (ng/ml), median (IQR) |  |  |  |  |  |  |  |  |  |  |  |
| Day 1 | 19.3 (16.3–24.8) | |  | 21.1 (16.5–24.5) | |  | -0.2 (-3.6 to 3.0) | |  |  | 0.95 |
| Day 3 | 24.9 (22.0–31.7) | |  | 21.3 (15.7–23.0) | |  | -5.9 (-10.6 to -2.2) | |  |  | 0.003 |
| Day 5 | 35.5 (25.5–42.3) | |  | 19.1 (16.9–23.4) | |  | -14.3 (-20.5 to -6.9) | |  |  | <0.001 |
| Day 7 | 31.5 (25.3–32.5) | |  | 19.8 (16.0–21.6) | |  | -11.0 (-14.8 to -5.6) | |  |  | 0.002 |
| MCP-1 (ng/ml), median (IQR) |  |  |  |  |  |  |  |  |  |  |  |
| Day 1 | 287.4 (256.1–347.1) | |  | 307.2 (257.9–343.3) | |  | -2.2 (-38.6 to 32.4) | |  |  | 0.95 |
| Day 3 | 347.5 (316.8–420.7) | |  | 309.2 (248.7–326.9) | |  | -63.3 (-113.6 to -23.1) | |  |  | 0.003 |
| Day 5 | 450.5 (343.2–523.3) | |  | 274.6 (251.5–320.0) | |  | -153.6 (-219.9 to -73.6) | |  |  | <0.001 |
| Day 7 | 407.6 (321.3–419.0) | |  | 282.1 (241.4–296.5) | |  | -121.2 (-159.2 to -43.4) | |  |  | 0.001 |
| Note: IQR denotes the interquartile range [median (25^th^ percentile–75^th^ percentile)]. The number of patients (for all variables): in the non-ICU subgroup: death group (first day n=4, third day n=4, and fifth day n=4); Survival group (first day n=42, third day n=42, fifth day n=42, and seventh day n=38), and the ICU subgroup: death group (first day n=15, third day n=11, fifth day n=8, and seventh day n=4); Survival group (first day n=37, third day n=37, fifth day n=37, and seventh day n=37). The difference was expressed as the median difference (Hodges–Lehmann estimate) and 95% confidence intervals.  Abbreviations: IL-6, interleukin-6; TNF-α, tumor necrosis factor alpha; IL-10, interleukin-10; IFN-γ, interferon-γ; IP-10, interferon gamma-induced protein 10; MIP-1α, macrophage inflammatory protein 1α; MCP-1, monocyte chemotactic protein 1. | | | | | | | | | | | |
